# Noise in NC-AFM measurements with significant tip–sample interaction

**DOI:** 10.3762/bjnano.7.181

**Published:** 2016-12-01

**Authors:** Jannis Lübbe, Matthias Temmen, Philipp Rahe, Michael Reichling

**Affiliations:** 1 Fachbereich Physik, Universität Osnabrück, Barbarastraße 7, 49076 Osnabrück, Germany; 2 Department of Physics and Astronomy, The University of Utah, 115 South 1400 East, Salt Lake City, UT 84112-0830, USA; 3 now at: School of Physics & Astronomy, The University of Nottingham, University Park, Nottingham NG7 2RD, UK

**Keywords:** amplitude noise, cantilever stiffness, closed loop, detection system noise, frequency shift noise, non-contact atomic force microscopy (NC-AFM), Q-factor, spectral analysis, thermal noise, tip–sample interaction

## Abstract

The frequency shift noise in non-contact atomic force microscopy (NC-AFM) imaging and spectroscopy consists of thermal noise and detection system noise with an additional contribution from amplitude noise if there are significant tip–sample interactions. The total noise power spectral density *D*^Δ^*^f^*(*f*_m_) is, however, not just the sum of these noise contributions. Instead its magnitude and spectral characteristics are determined by the strongly non-linear tip–sample interaction, by the coupling between the amplitude and tip–sample distance control loops of the NC-AFM system as well as by the characteristics of the phase locked loop (PLL) detector used for frequency demodulation. Here, we measure *D*^Δ^*^f^*(*f*_m_) for various NC-AFM parameter settings representing realistic measurement conditions and compare experimental data to simulations based on a model of the NC-AFM system that includes the tip–sample interaction. The good agreement between predicted and measured noise spectra confirms that the model covers the relevant noise contributions and interactions. Results yield a general understanding of noise generation and propagation in the NC-AFM and provide a quantitative prediction of noise for given experimental parameters. We derive strategies for noise-optimised imaging and spectroscopy and outline a full optimisation procedure for the instrumentation and control loops.

## Introduction

Non-contact atomic force microscopy (NC-AFM) [1–2] is an unmatched surface science tool, especially when it comes to studying non-conducting surfaces [3–4], to map sub-molecular structures [5] or to measure forces [6] and force fields [7] with highest resolution. The primary imaging signal in NC-AFM is the frequency shift Δ*f* of a probe resonator carrying a tip interacting with the sample surface [2], typically a cantilever, a tuning fork, or a needle sensor [8].

The resolution of force measurements is limited by the noise in the frequency shift signal [9–10], which strongly depends on the noise floor of the detection system, the frequency response of the frequency demodulator (mostly a phase-locked loop detector, PLL), cantilever properties and ultimately thermal noise [11]. The footing of our work are these precursor studies, and the rigorous system analysis introduced by Polesel-Maris et al. [12], showing that the frequency shift noise at close tip–sample distance is increased due to a coupling of the phase-locked loop with the amplitude and the distance control loops.

While noise in the amplitude control loop itself is essentially independent of the frequency shift noise without tip–sample interaction, amplitude and topography feedback loop noise are coupled into the frequency shift noise in the presence of tip–sample forces [12]. Ultimately, the noise in the frequency shift signal determines the base performance of all downstream processing such as the topography signal or the Kelvin probe force signal [13].

Here, we use the formalism derived by Polesel-Maris et al. [12], introduce realistic transfer functions for the control electronics, cantilever properties and tip–sample interaction, to quantitatively determine the frequency shift noise in the presence of significant tip–sample interaction, to derive predictions for noise spectra and to correlate them with experimental data obtained under realistic measurement conditions. We find excellent agreement between simulated and experimental results for noise in a cantilever-based NC-AFM with optical beam-deflection and measurements performed in an ultra-high vacuum environment, where the cantilever Q-factor is close to the intrinsic value *Q*_0_ [14–15]. Our analysis can, however, be applied to any NC-AFM detection scheme and sample environment, specifically also to measurements in liquids where signal-to-noise-ratio considerations play a paramount role [16–18]. From our findings, we derive a general strategy for adjusting instrumental settings and control loops for noise-optimised operation. A full glossary of all of these settings and further quantities relevant in this context are compiled in appendix A.

Our analysis is based on four fundamental steps: First, the cantilever oscillation amplitude is determined precisely by calibrating the voltage signal proportional to the cantilever displacement with a method described in detail elsewhere [19]. This yields the detection sensitivity 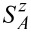 (see also Supplementary Information section 1 of [11]). Second, 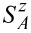 is used to convert the displacement noise voltage signal into the displacement noise quantities, namely the displacement noise power spectral density 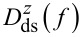 of the detection system (frequently referred to as the noise floor) and the thermal noise power spectral density 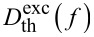 [11]. Note that the latter cantilever thermal excitation noise contribution can be predicted from the oscillator properties and temperature [11]. Third, the frequency response *H*_filter_ of the PLL system is used for describing the propagation of noise from the cantilever oscillation to the frequency shift signal at the output of the frequency demodulator. This frequency response function strongly depends on the PLL filter settings [11] and will here be modelled for a typical experimental setup described in section “Noise propagation model” and appendix C. Fourth, we determine the explicit frequency response functions *H_A_* and *H_z_* of the amplitude and topography control loops, respectively. This allows an adjustment of the amplitude control loop and the frequency response of the PLL prior to the measurement when tip–sample interaction is absent (i.e., with the tip retracted). The frequency response of the distance control loop, however, inherently depends on the tip–sample interaction which is, in turn, preset by the *z*-position along the force–distance curve [12]. Therefore, this control loop needs adjustment under conditions of the envisaged measurement.

After describing experimental methods and procedures in section “Experimental”, we introduce the NC-AFM model used to simulate noise generation and propagation in section “Noise propagation model”. In section “Tip–sample interaction”, we then discuss the implications of the tip–sample interaction on the coupling of control loops. After a check of validity and consistency of the model by testing simulation results against measurements for the case of absent tip–sample interaction in section “Noise with negligible tip–sample interaction”, we systematically explore cases with significant tip–sample interaction in section “Noise with significant tip–sample interaction”. The investigation comprises measurements and simulations for scanning the surface at a constant tip–sample distance (constant-height mode) as well as with the frequency shift kept at a certain value by the *z*-control loop (topography mode). For the simulations, the filter settings of the control loops are varied over ranges of values typically present in experiments, and an artificial but realistic model potential is used for the tip–sample interaction. We validate the noise model including tip–sample interaction and describe a rational procedure for choosing system parameters for noise-optimised measurements in section “Conclusions and system optimisation”. All equations within this work are written using power spectral densities *D^X^* for the quantity *X*, while simulated and experimental results are described in terms of amplitude spectral densities 
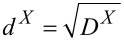
.

## Experimental

All experiments are performed using a commercial NC-AFM system (UHV 750 variable temperature STM/AFM, RHK Technology, Inc., Troy, MI, USA) operated at room temperature and employing the beam-deflection method to measure the cantilever displacement. Tip positioning and approach is accomplished by the SPM 100 control system (RHK Technology, Inc.). For this and all other instruments we introduce scaling factors to convert voltage signals delivered by the instruments to physical units. The detection sensitivity 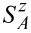 for the herein presented data is determined to 52.5 nm/V from an amplitude calibration (see [19] and Supporting Information of [11]). A PLLpro2 control system (RHK Technology, Inc., Troy, MI, USA) is used for frequency demodulation and amplitude stabilisation. This control system encodes the frequency shift Δ*f* in volts using *S*^Δ^*^f^* = −30 Hz/V. For the distance control loop, we employ a digital PI controller of the HF2LI device (Zurich Instruments AG, Zürich, Switzerland) as this instrument provides loop filters with well-defined characteristics. Noise measurements at the Δ*f* and amplitude outputs of the PLL system as well as at the topography output of the distance controller are performed using a SR770 spectrum analyser (Stanford Research Systems, Inc., Sunnyvale, CA, USA). The topography signal is scaled using the sensitivity 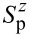 = 9.36 nm/V for the scanner *z*-piezo response. This value was determined from measuring step heights on CaF_2_ surfaces.

The cantilever is a commercial silicon cantilever (type PPP-NCH, Nanoworld AG, Neuchâtel, Switzerland) with an eigenfrequency of *f*_0_ = 305337.6 Hz at room temperature and a quality factor of *Q*_0_ = 43900 determined as described elsewhere [14]. The noise floor is determined to 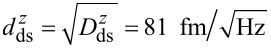 and the modal stiffness of the cantilever [20] to *k*_0_ = 32.4 N/m from a measurement of the thermally excited cantilever oscillation [11] with the spectrum analyser of the HF2LI device. The cantilever oscillation is stabilised at an amplitude of *A* = 13.6 nm, which corresponds to an amplitude of *A_z_* = *A* cos(θ) = 12.6 nm perpendicular to the surface due to the inclination of θ = 22.5° between cantilever and sample surface given by the cantilever mount. These experimental parameters are used in all simulations presented within this work.

The tip–sample interaction modelled by the parameter β_ts_ (see section “Tip–sample interaction”) is derived from a measured Δ*f*(*z*_p_) curve shown in Figure 4). Here, Δ*f* is plotted against the piezo position *z*_p_. Depending on the operation mode (constant-height or topography), the parameter β_ts_ can be obtained by using either the frequency shift set-point Δ*f*_set_ for the topography feedback or by the average frequency shift 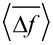 measured at the tip–sample distance *z*_p_ with deactivated topography feedback loop.

For the numerical evaluation of signal vs time traces and noise spectra, the explicit frequency response functions and system parameters for our experimental setup are used; all frequency response functions are listed in the appendix and the implementation in MATLAB is available in Supporting Information File 1. This approach enables a numeric evaluation in absolute physical units and, therefore, allows the direct comparison between experiment and our model.

## Results and Discussion

### Noise propagation model

**Figure 1:**
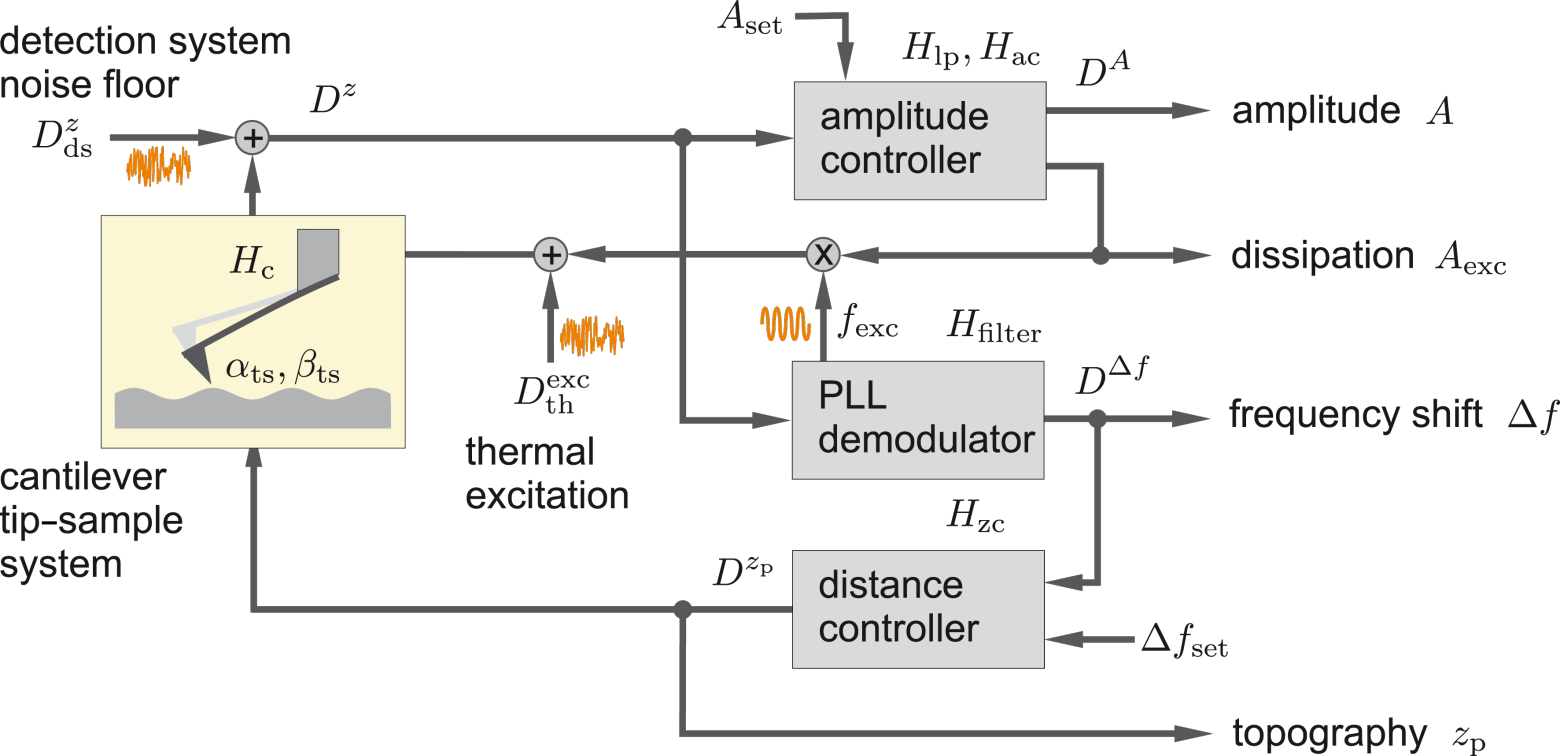
Schematic representation of functional elements of an NC-AFM described by transfer functions *H*_y_. Quantities *D*^X^ denote noise power spectral densities of the signal X. Symbols “+” and “×” denote entry points of noise and entanglement of signals, respectively.

Figure 1 illustrates the signal and noise propagation in a typical NC-AFM setup. The cantilever is excited by a drive signal with frequency *f*_exc_ and amplitude *A*_exc_. Additionally, the cantilever experiences an excitation due to thermal noise expressed by the power spectral density 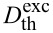 = 2*k*_B_*T*/(*k*_0_*Q*_0_π*f*_0_). The cantilever responds to these excitations with an oscillation of amplitude *A* dictated by the cantilever response function *H*_c_(*f*). This cantilever oscillation is measured as the cantilever displacement signal. Noise contributions in this signal are described in frequency space by the thermal noise displacement power spectral density 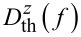 and by the detection system noise power spectral density 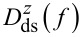, the latter caused by the electronic detection system [11]. The sum of the detection system noise power spectral density 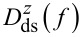 and the thermal displacement noise power spectral density 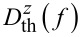 yields the total displacement noise power spectral density *D^z^*(*f*).

The cantilever displacement signal is fed into both, the amplitude controller and the PLL demodulator. The amplitude controller measures the oscillation amplitude *A* typically using a root-mean-square algorithm or lock-in detection and adjusts the excitation amplitude *A*_exc_ to keep the oscillation amplitude *A* at the set-point *A*_set_. The amplitude measurement includes a low-pass filter with the response function *H*_lp_(*f*), while the amplitude controller is described by the frequency response *H*_ac_(*f*). Noise in the amplitude signal is characterised by the amplitude noise power spectral density *D^A^*(*f*).

The PLL demodulator determines the frequency shift Δ*f* = *f*_r_− *f*_0_, which is the difference between the cantilever resonance frequency *f*_r_ in the presence of tip–sample interaction and the cantilever eigenfrequency *f*_0_. Furthermore, the demodulator provides the cantilever excitation signal with frequency *f*_exc_ that is nominally identical to the current resonance frequency *f*_r_ of the cantilever. The frequency shift noise power spectral density *D*^Δ^*^f^*(*f*_m_) depends on the filter and loop settings of the PLL demodulator expressed by its frequency response function *H*_filter_(*f*_m_), where *f*_m_ represents the frequencies of the modulation side bands measured relative to the resonance frequency *f*_r_ [11]. Thus, the cantilever excitation signal contains noise from both, the PLL and the amplitude controller.

The frequency shift signal is fed into the distance controller, which adjusts the tip–sample distance to maintain a frequency shift equal to the set-point Δ*f*_set_. The tip–sample distance is expressed as the position *z*_p_ of the *z*-piezo (see below in Figure 3) and is in this context commonly referred to as the topography signal. The distance-dependent frequency shift Δ*f*(*z*_p_) is governed by the details of the tip–sample interaction forces, and is herein for a few specific tip–sample distances characterised by the two parameters α_ts_(*z*_p_) and β_ts_(*z*_p_) as described in section “Tip–sample interaction”. These parameters determine how fluctuations in the oscillation amplitude and the tip–sample distance are coupled into the frequency shift signal.

**Figure 2:**
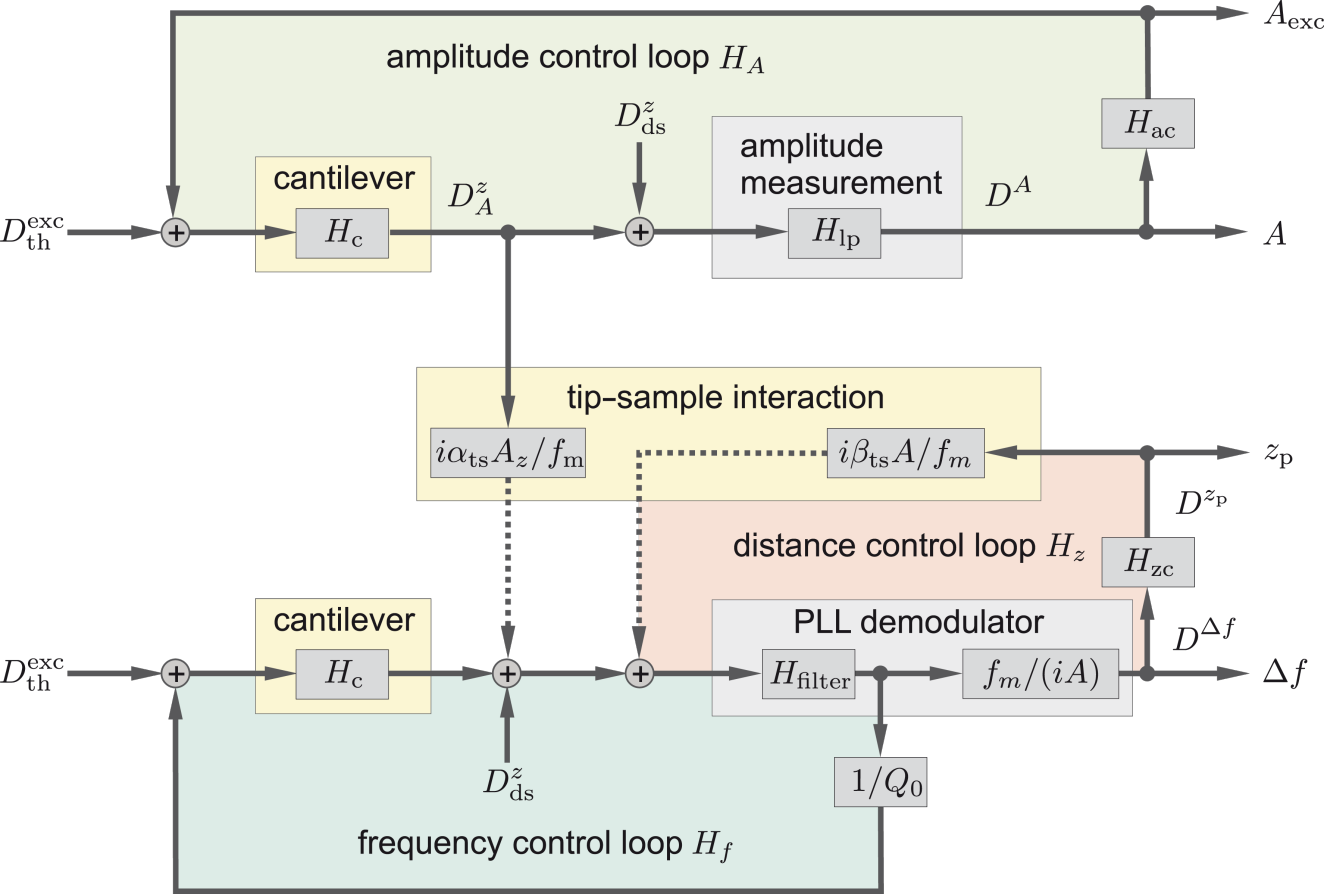
Model for signal and noise propagation in an NC-AFM, highlighting the tip–sample interaction, PLL demodulator and control loops. Signal paths indicated by dotted lines are only relevant for the case of significant tip–sample interaction.

The noise propagation model used for our simulations is based on the approach introduced in [12] and sketched in Figure 2. In contrast to the NC-AFM functional scheme shown in Figure 1, here we focus on the noise signal paths and transfer blocks relevant to the noise propagation. Furthermore, we investigate amplitude noise and frequency shift noise in two separate loops that are coupled via the tip–sample interaction. Effectively, this approach splits the signal into a purely amplitude-modulated component (controlled by the amplitude control loop) and a signal with pure frequency (or phase) modulation (controlled by the frequency control loop including the PLL) [12]. This separation stems from the small intermixing strength of the two modulations, and will be justified here based on experimental evidence.

In the **amplitude control loop** (top part of Figure 2), the cantilever displacement signal contains amplitude fluctuations described by the noise power spectral density 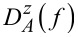 and the detection system adds the noise floor 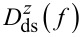, yielding the measured displacement noise *D^z^*(*f*). The amplitude signal *A* follows from the displacement signal, which is then low-pass-filtered as described by the transfer function *H*_lp_, and finally contains noise with the amplitude power spectral density noise *D^A^*(*f*_m_). This signal is fed into the amplitude controller described by the transfer function *H*_ac_, generating the excitation signal amplitude *A*_exc_. The amplitude control loop is closed by feeding this signal to the cantilever. Note that with closing the loop, a fraction of the noise *D^A^*(*f*_m_) is fed back to the cantilever, added to the thermal noise 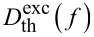 and filtered by the narrowband cantilever response function *H*_c_(*f*).

In the **frequency control loop** (bottom part of Figure 2), the measured cantilever displacement signal is fed into the PLL demodulator yielding the frequency shift signal Δ*f* as well as the excitation signal for the cantilever in the feedback path. The control loop within the PLL demodulator (not shown in Figure 2) is discussed in appendix C.2. In this frequency control loop feedback path, displacement noise propagating from the PLL to the cantilever excitation is weighted by the reciprocal of the quality factor *Q*_0_. This factor defines the ratio of the cantilever excitation signal to the oscillation amplitude at the cantilever resonance if neither amplitude noise nor amplitude disturbances are present, i.e., in absence of tip–sample interactions. The sum of excitation signal noise and thermal excitation noise 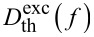 is band-pass-filtered by the cantilever response function *H*_c_(*f*) and added to the detection system noise floor 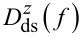. The loop is closed by feeding this signal into the PLL. In the case of negligible tip–sample interaction, the noise in the frequency control loop is virtually independent from the settings of the other control loops shown in Figure 2, although we note that a coupling may become apparent if either of the loops is operated in an unstable ringing configuration. If significant tip–sample interaction is present, two more signals, one from the amplitude and a second from the distance control loop, are added before feeding the signal into the PLL demodulator as described below.

The **distance control loop** employs a controller with transfer function *H*_zc_ to regulate the frequency shift Δ*f* from the PLL by adjusting the piezo position *z*_p_. The slope β_ts_ = ∂Δ*f*/∂*z* of the frequency shift vs distance curve Δ*f*(*z*) models the tip–sample interaction and is usually a non-linear function of *z*_p_. The frequency shift noise *D*^Δ^*^f^*(*f*_m_) is converted to topography noise 
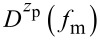
 by the action of the distance controller with transfer function *H*_zc_. The topography noise is scaled by the tip–sample interaction transfer function *i*β_ts_*A*/*f*_m_ and added to other noise contributions at the PLL demodulator input. The loop is closed across the PLL.

The **coupling** between the amplitude and the frequency control loops, which exists in the presence of significant tip–sample interaction, is modelled by a transfer function *i*α_ts_*A*/*f*_m_ with α_ts_ = ∂Δ*f*/∂*A*, acting on the amplitude noise 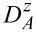. The resulting noise is one of the contributions at the PLL demodulator input and increases the frequency shift noise.

### Tip–sample interaction

The tip–sample interaction closes the distance control loop and it couples the amplitude control loop with the frequency control loop. Both connections can significantly increase the noise in the frequency shift Δ*f* output signal compared to the case of negligible tip–sample interaction.

The transfer of fluctuations from the piezo position *z*_p_ into the cantilever deflection signal by the distance control loop (see Figure 2) is described by the parameter β_ts_. This parameter is defined as the gradient of the frequency shift signal Δ*f* with respect to the tip–sample distance *z*_ts_ (see [12] and appendix D):

[1]
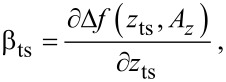


[2]
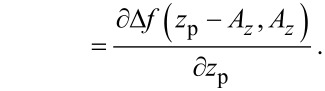


**Figure 3:**
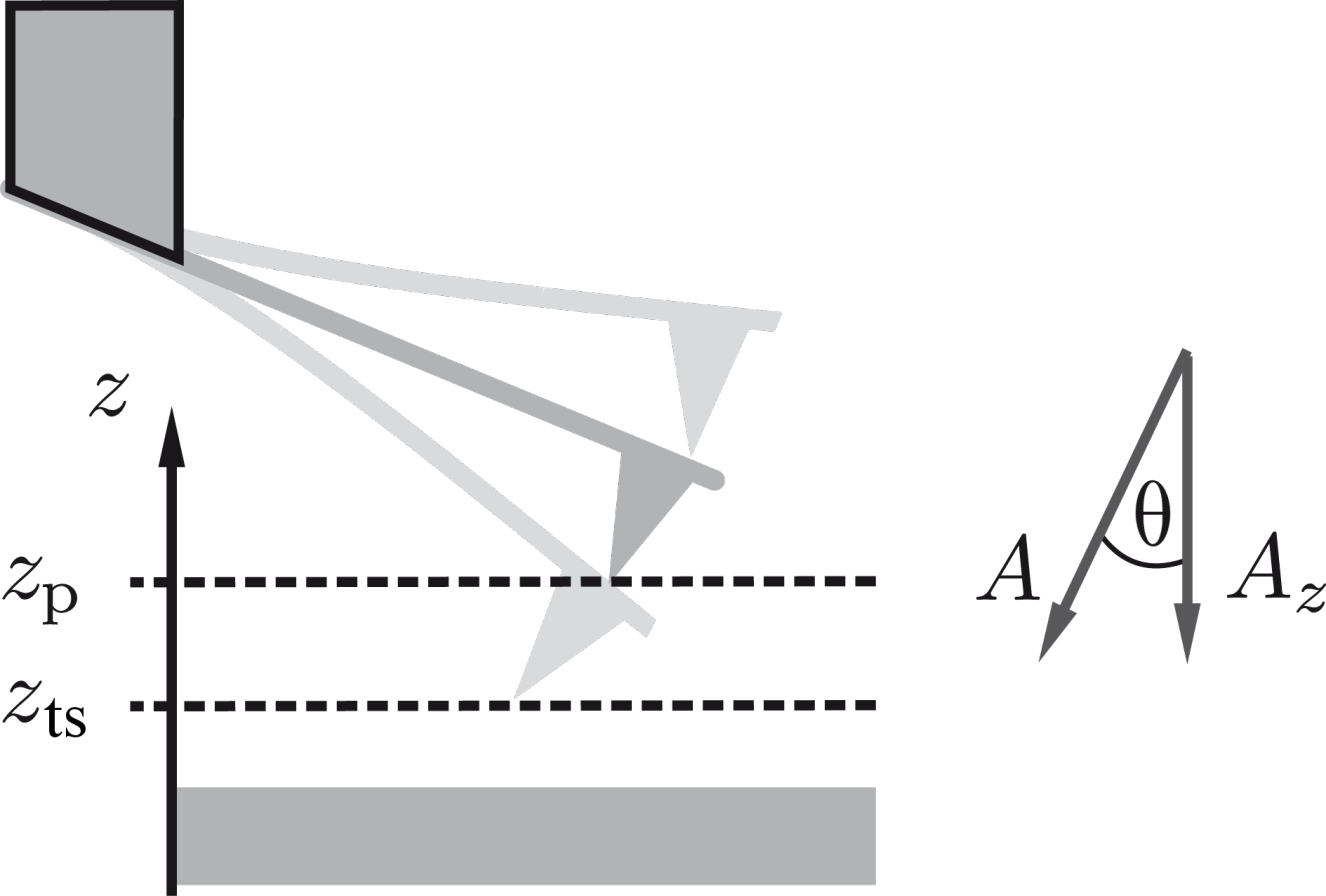
Relations between the piezo position *z*_p_ (tip position for resting cantilever), the lower turning point *z*_ts_ of the cantilever oscillation and the oscillation amplitude *A* as well as its projection *A_z_* on the sample surface normal.

**Figure 4:**
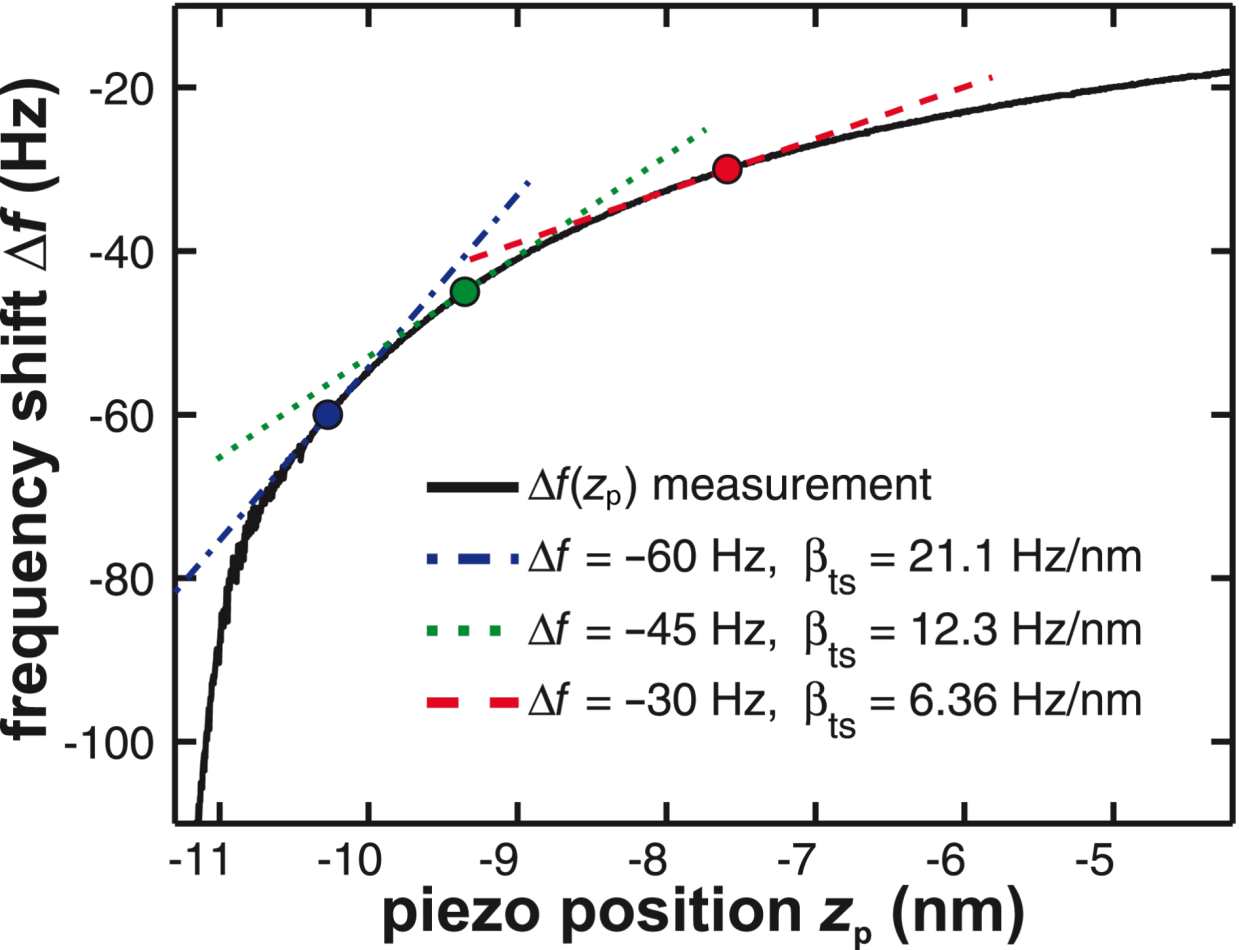
Determination of the tip–sample interaction parameter β_ts_ from the slope of a measured Δ*f*(*z*_p_) curve. Frequency shift data are plotted as a function of the *z*-piezo position *z*_p_.

The parameter β_ts_ can be parameterised by either the *z*-position of the lower turning point *z*_ts_ (Equation 1) or using the piezo position *z*_p_ (Equation 2), which is the centre position of the cantilever oscillation (see Figure 3). We explicitly include the amplitude dependency on the frequency shift Δ*f* by including the oscillation amplitude component *A_z_* perpendicular to the sample surface. This dependency follows from the convolution of the interaction force with the weighting function due to the cantilever oscillation. For large oscillation amplitudes *A_z_*, the functional dependence 
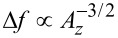
 has been found [21]. Hence, for a small variation δ*z*_p_ of the *z*_p_ position, β_ts_ can straightforwardly be determined from the slope of the Δ*f*(*z*_p_) curve at the working point as illustrated by the model curve in Figure 4. Obviously, β_ts_ strongly varies as a function of *z*_p_.

The parameter α_ts_ describes the transfer of cantilever deflection noise 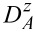 from the amplitude control loop into deflection noise in the frequency control loop via two mechanisms. First, a variation δ*A* in the amplitude changes the weighting function in calculating the frequency shift from the cantilever oscillation [21] and, thus, the magnitude of the resulting Δ*f*. Second, the variation leads to a shift of the lower turning point *z*_ts_, bringing the sensor into a different tip–sample interaction regime. The coupling parameter α_ts_ is defined by [12]

[3]
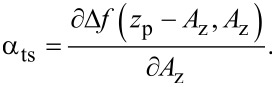


For the experimental conditions within this work (see appendix D), small fluctuations δ*A_z_* of the oscillation amplitude have the same effect as a small fluctuation δ*z* in the center position, namely δ*z* = −δ*A_z_*. Therefore, we use the approximation

[4]
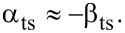


Further details on the relation between α_ts_ and β_ts_ assuming a model potential for the tip–sample interaction are provided in appendix D. Short-range forces acting between the probing tip and the sample surface are of primary relevance for our discussion as they typically exhibit strong gradients. Thus, the coupling strongly increases with increasing interaction when the tip is closely approached to the surface.

### Noise with negligible tip–sample interaction

We first analyse noise in the frequency shift Δ*f* and amplitude *A* channel for the case of negligible tip–sample interaction (α_ts_ = β_ts_ = 0) to check for consistency with previous simulations [12] as well as experimental results [11]. In respective experiments, we prepare this situation by retracting the tip several tens of nanometres from the sample surface.

The frequency shift noise power spectral density 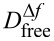 strongly depends on the PLL demodulator parameters [11] and is explicitly given by evaluating the frequency control loop in Figure 2 (see appendix B)

[5]
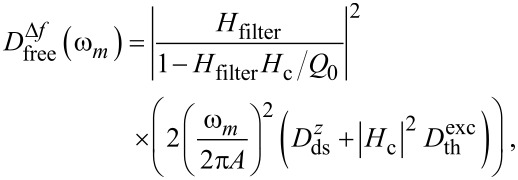


where the system parameters are those introduced in Figure 2. We use the explicit description of our experimental system (see appendix C for the individual frequency response functions) to numerically evaluate Equation 5. Note that the comparison of simulated noise spectra with experimental data is based on these system parameters and not on fitting.

The amplitude noise power spectral density *D^A^* is calculated from evaluating the amplitude control loop in Figure 2

[6]
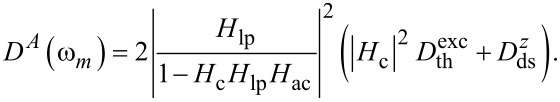


We use the explicit system parameters for our experimental setup to evaluate Equation 6 numerically in absolute physical units.

**Figure 5:**
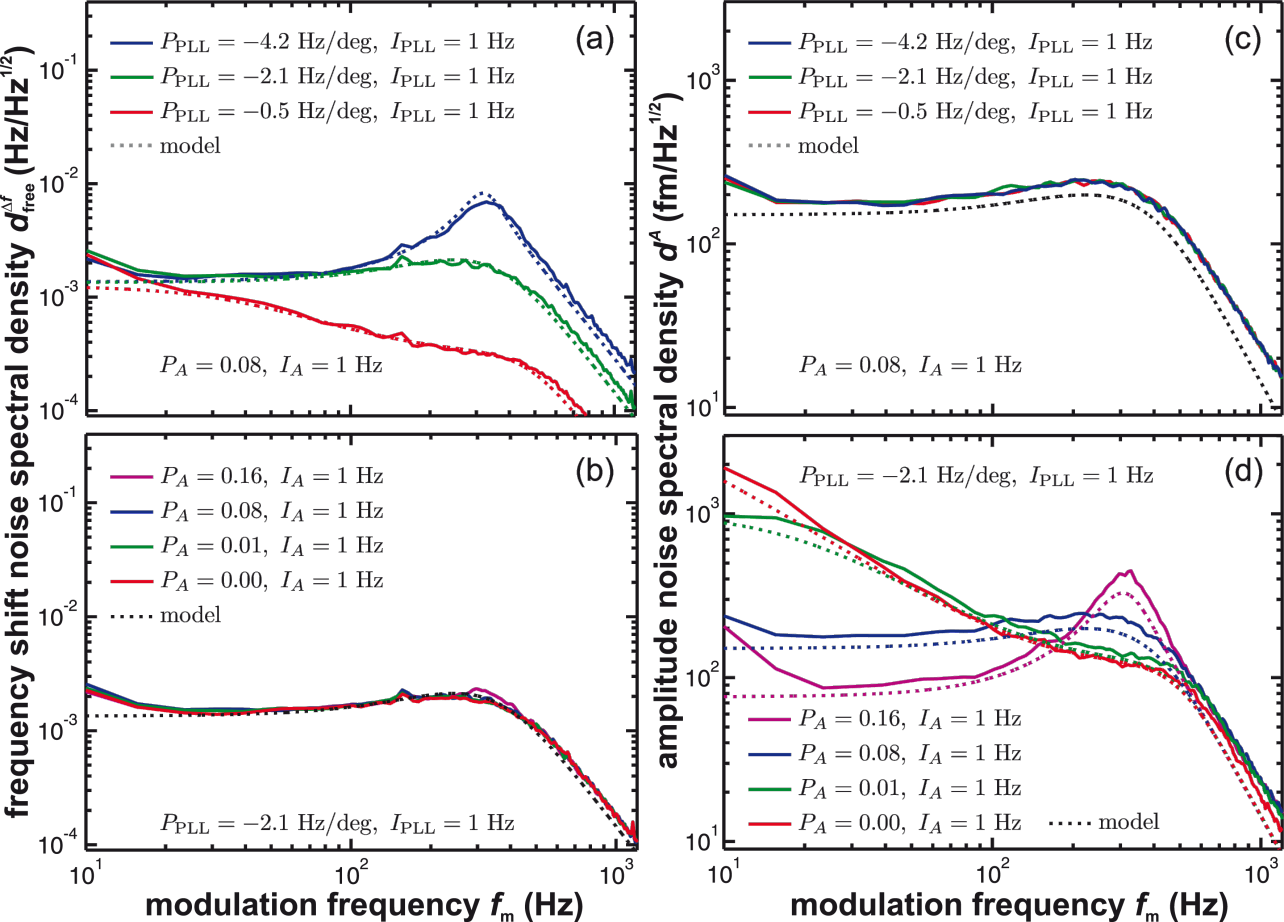
Measured noise spectral density (solid lines) of (a, b) the frequency shift signal and (c, d) the amplitude signal for a variation of the proportional loop gain settings *P*_PLL_ and *P_A_* of the PLL and amplitude control loop, respectively. The integral cutoff of the PLL loop (*I*_PLL_) and of the amplitude loop (*I*_A_) are each held constant. The tip is retracted from the surface for the measurements. Model calculations (dotted lines) based on Equation 5 and Equation 6 are performed assuming negligible tip–sample interaction. The loop filter *H*_lp_ has a 3rd-order Butterworth characteristics with a cutoff frequency of *f*_c_ = 500 Hz, all other quantities are explicitly given in appendix C.

In Figure 5, we compile measurements (solid lines) and simulations (dotted lines) for the frequency shift noise amplitude spectral density 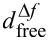 (panels (a) and (b)) and the amplitude noise amplitude spectral density *d^A^* (panels (c) and (d)). Panels (a) and (c) represent results for optimised amplitude loop gain settings while varying the PLL parameters. In contrast, panels (b) and (d) show results for optimised PLL parameters while varying the amplitude gain settings. In all data, the amplitude control loop filter *H*_lp_ has a 3rd-order Butterworth characteristics with a cutoff frequency of *f*_c_ = 500 Hz.

Figure 5a demonstrates the low-pass filter characteristics of the PLL in the case of excessive filtering (low P-gain), optimum operating conditions (optimum P-gain) and gain peaking (high P-gain), respectively. The optimum frequency response is determined as described in appendix C, yielding optimum parameters of *P*_PLL_ = −2.1 Hz/deg and *I*_PLL_ = 1 Hz. Note that the PLL frequency response does not depend on the cantilever parameters. Thus, it can be optimised for the desired detection bandwidth by only considering the cantilever parameters. In contrast, the frequency shift noise at the PLL output generated by the frequency control loop depends on cantilever properties and several other system parameters including the PLL settings. The amplitude noise presented in Figure 5c is independent of the PLL loop settings and, similarly, the frequency shift noise shown in Figure 5b is independent of the amplitude loop settings, clearly demonstrating that the amplitude and frequency control loops are not coupled unless the PLL is operated in an unstable regime.

The spectral behaviour of the amplitude noise *d^A^* upon changing the amplitude control loop settings is slightly different from the behaviour observed in the frequency shift noise 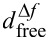 upon changing the frequency control loop gain settings as demonstrated in Figure 5a and Figure 5d. When increasing the P-gain of the amplitude control loop, the noise in the low-frequency region decreases while in this case a peak around a frequency of about 300 Hz develops. If the amplitude control loop is disabled (*P_A_* = 0, red line), the noise spectral density becomes large in the low-frequency region as predicted by the simulation (dotted line). Thus, an activated amplitude control loop effectively compensates low-frequency noise in the amplitude signal. Optimum performance of this loop is obtained for the parameters *P_A_* = 0.08 and *I_A_* = 1 Hz using the criteria introduced in appendix C.

In conclusion, we find excellent quantitative agreement between simulated and experimental data for various settings of the amplitude and frequency control loop. The independence of the frequency shift noise (amplitude noise) upon changing the amplitude (frequency) control loop settings, respectively, clearly demonstrates the validity of separating the system in these two control loops as depicted in Figure 2 for the case of negligible tip–sample interaction.

### Noise with significant tip–sample interaction

Realistic NC-AFM imaging or force mapping experiments are performed at a small tip–sample distance, or even in the repulsive regime [22], where large gradients of the tip–sample force generate strong gradients in the frequency shift signal. We now extend Equation 5 and Equation 6 to include the additional noise contributions predicted by [12] and our system model in Figure 2.

The noise power spectral density of the cantilever oscillation amplitude 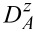 is not directly accessible experimentally, but can be introduced by analysing the amplitude control loop (see Figure 2)

[7]
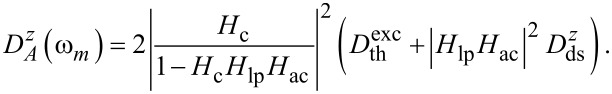


The quantity 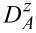 itself is not affected by the tip–sample interaction. However, due to the coupling characterised by the parameter α_ts_, the noise spectral density 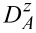 propagates into the frequency control loop, yielding a significant contribution to the frequency shift noise. From including this contribution in the control loop diagram of Figure 2, we find the frequency shift noise power spectral density *D*^Δ^*^f^* as

[8]
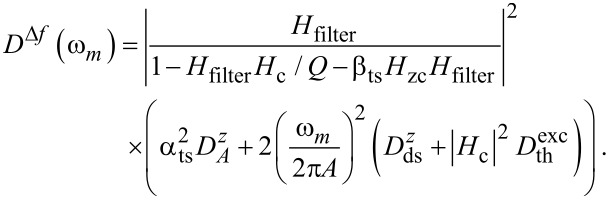


Following the approach from the previous section, we use the explicit system parameters and system-specific transfer functions (given in appendix C) to numerically evaluate Equation 8 for comparison with the experimental data.

**Figure 6:**
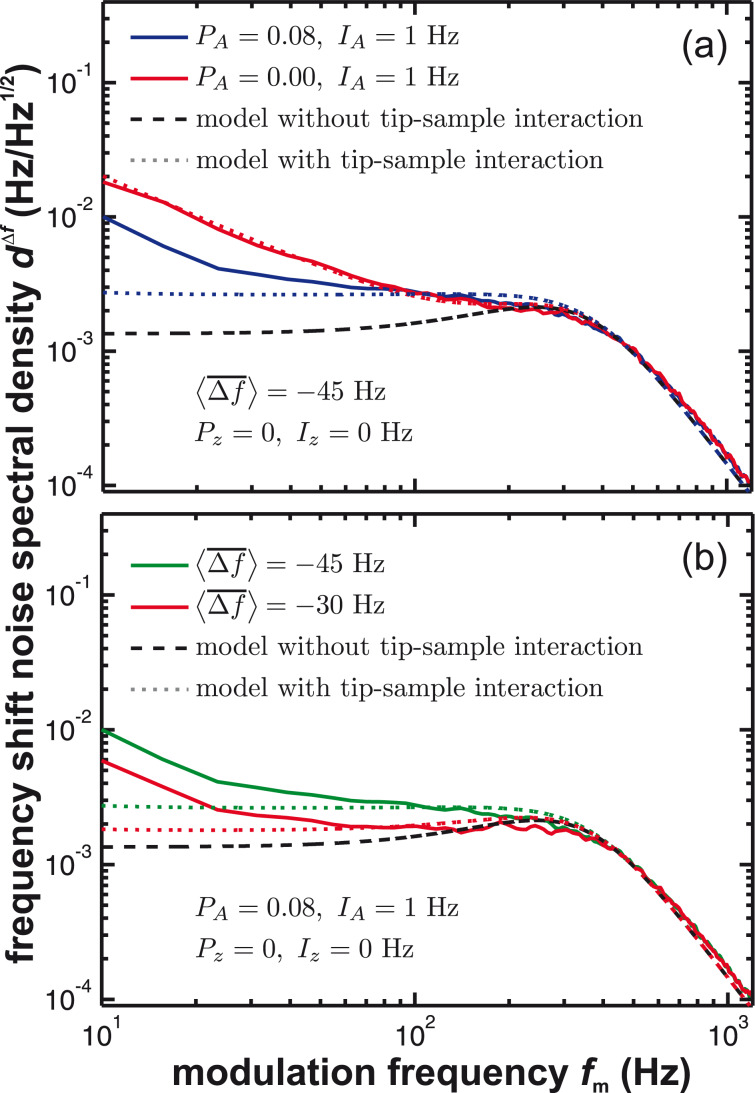
Frequency shift noise spectral density *d*^Δ^*^f^* for the case of significant tip–sample interaction measured in the constant height mode (*P_z_* = 0, *I_z_* = 0 Hz) in dependence on (a) the amplitude control loop settings and (b) the tip–sample distance parametrised via the averaged frequency shift 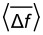. Measured curves (solid lines) are compared to model predictions including tip–sample interaction (dotted lines, Equation 8) and to the model without tip–sample interaction (dashed lines, Equation 5). The loop filter *H*_lp_ has a 3rd-order Butterworth characteristics with a cutoff frequency of *f*_c_ = 500 Hz.

First, we investigate NC-AFM experiments performed in the constant-height mode, where the tip is in close proximity to the sample surface with the distance control loop disabled, modelled here by setting *H*_zc_ = 0. Measurements (solid lines) and corresponding simulations using Equation 8 (including tip–sample interaction, dotted lines) and Equation 5 (without tip–sample interaction, dashed lines) of the frequency shift noise spectral density *D*^Δ^*^f^* are reproduced in Figure 6. Measurements and simulations are performed with enabled (*P_A_* = 0.08) and disabled (*P_A_* = 0) amplitude control loop as shown in Figure 6a and at two tip–sample distances characterised by the averaged frequency shift 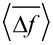 as shown in Figure 6b. The increase in the spectral noise at low frequencies in Figure 6a indicates contributions from the cantilever amplitude noise coupling into the frequency shift signal via the tip–sample interaction. Despite some discrepancy at very low frequencies also observed for the case of negligible tip–sample interaction (see Figure 5), we find a good agreement between prediction and experimental results. Here, we can only speculate that the low-frequency deviation is caused by mechanical instabilities within the system, or by instabilities within the piezoelectric excitation system. For example, low-frequency noise has been observed when using photothermal excitation [23].

Disabling the amplitude control loop results in a strong increase of low frequency noise compared to operation with engaged amplitude control using optimum parameters (see previous section and appendix C). The amplitude control loop effectively reduces the frequency shift noise by its negative feedback. Furthermore, we observe an increase of the frequency shift noise *D*^Δ^*^f^* for stronger tip–sample interaction (see Figure 6b) due to a strong coupling described by an increase of α_ts_ at smaller tip–sample distances.

Second, we investigate the frequency shift and topography noise in the commonly used constant frequency-shift mode where the tip–sample distance is adjusted by the distance control loop to keep the frequency shift at the set-point Δ*f*_set_. The topography noise spectral density 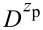 is obtained by applying the frequency response *H*_zc_ of the distance controller to the frequency shift noise *D*^Δ^*^f^* (see Figure 2)

[9]
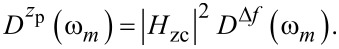


**Figure 7:**
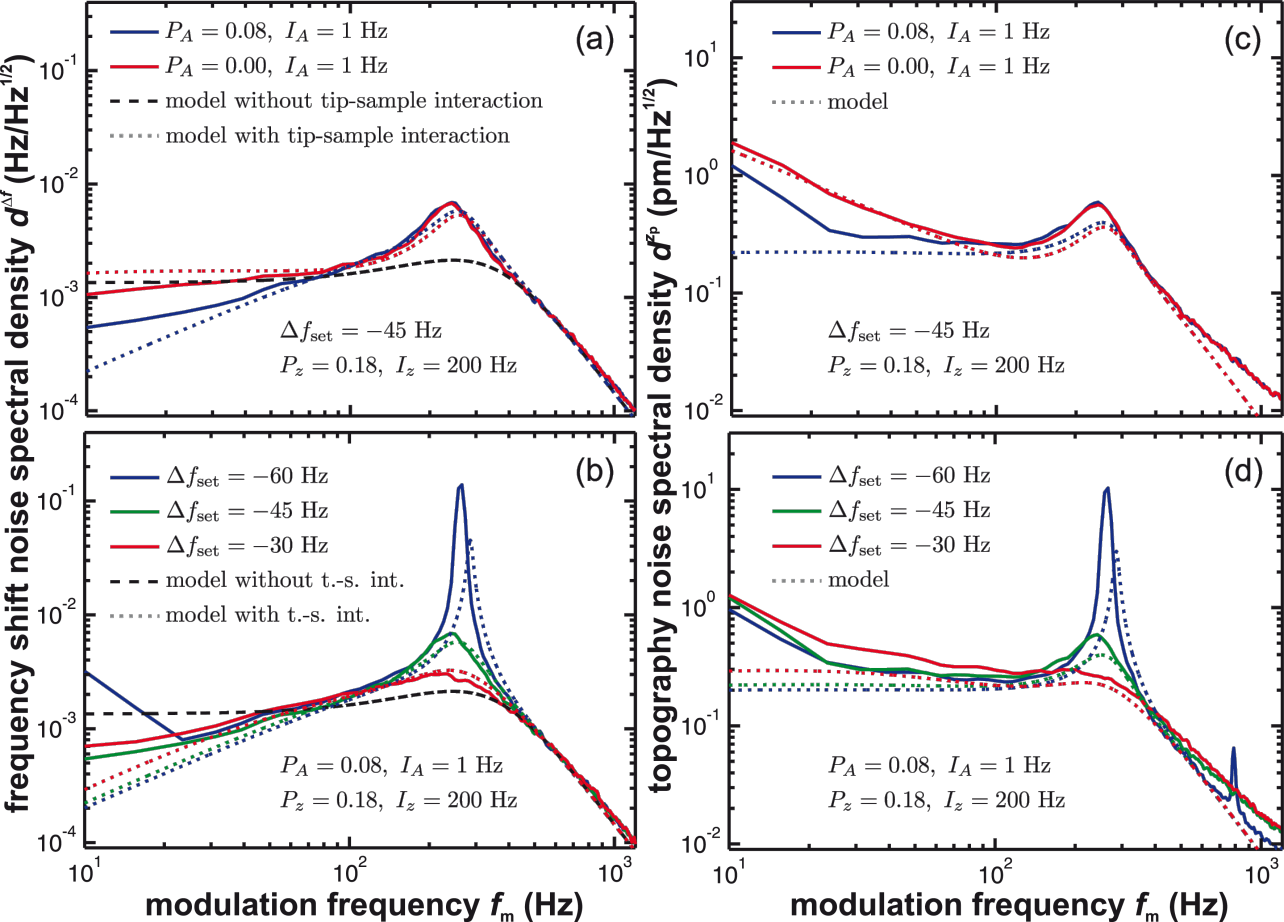
(a, b) Frequency shift noise spectral density *d*^Δ^*^f^* and (c, d) topography noise spectral density 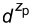 with tip–sample interaction using the constant frequency-shift mode in dependence on the amplitude control loop settings and the tip–sample distance defined by the frequency shift set-point Δ*f*_set_. Measured curves (solid lines) are compared to model predictions including tip–sample interaction (dotted lines, Equation 8 and Equation 9) and without tip–sample interaction (dashed lines, Equation 5). The loop filter *H*_lp_ has a 3rd-order Butterworth characteristics with a cutoff frequency of *f*_c_ = 500 Hz.

Figure 7 shows the measured frequency shift (panels (a, b)) and topography (panels (c, d)) noise in the presence of the activated distance control loop (solid lines). These experimental data are compared to simulation results based on Equation 8 including tip–sample interaction (dotted lines) and Equation 5 without tip–sample interaction (dashed lines).

Generally, we observe an increase of noise power in the frequency range from 200 to 300 Hz, where the apparent peaking firsthand appears to be independent from the amplitude control loop settings. Engaging the amplitude control loop (Figure 7a and Figure 7c) results in a reduction of noise in the low-frequency regime as observed in the constant-height measurement mode. However, the active loop has a marginal influence on the peaking in the 200–300 Hz region. In contrast, the appearance of this peak strongly depends on the frequency shift set-point: The peak height increases with increased frequency shift set-point Δ*f*_set_ (Figure 7b and Figure 7d). Interestingly, with activated distance control, the noise level in the low-frequency range may even fall below the values observed without tip–sample interaction. This demonstrates that the distance control loop is effectively able to compensate some of the low-frequency thermal noise by a distance adjustment and directly suggests an optimum frequency response as outlined in appendix C.

**Figure 8:**
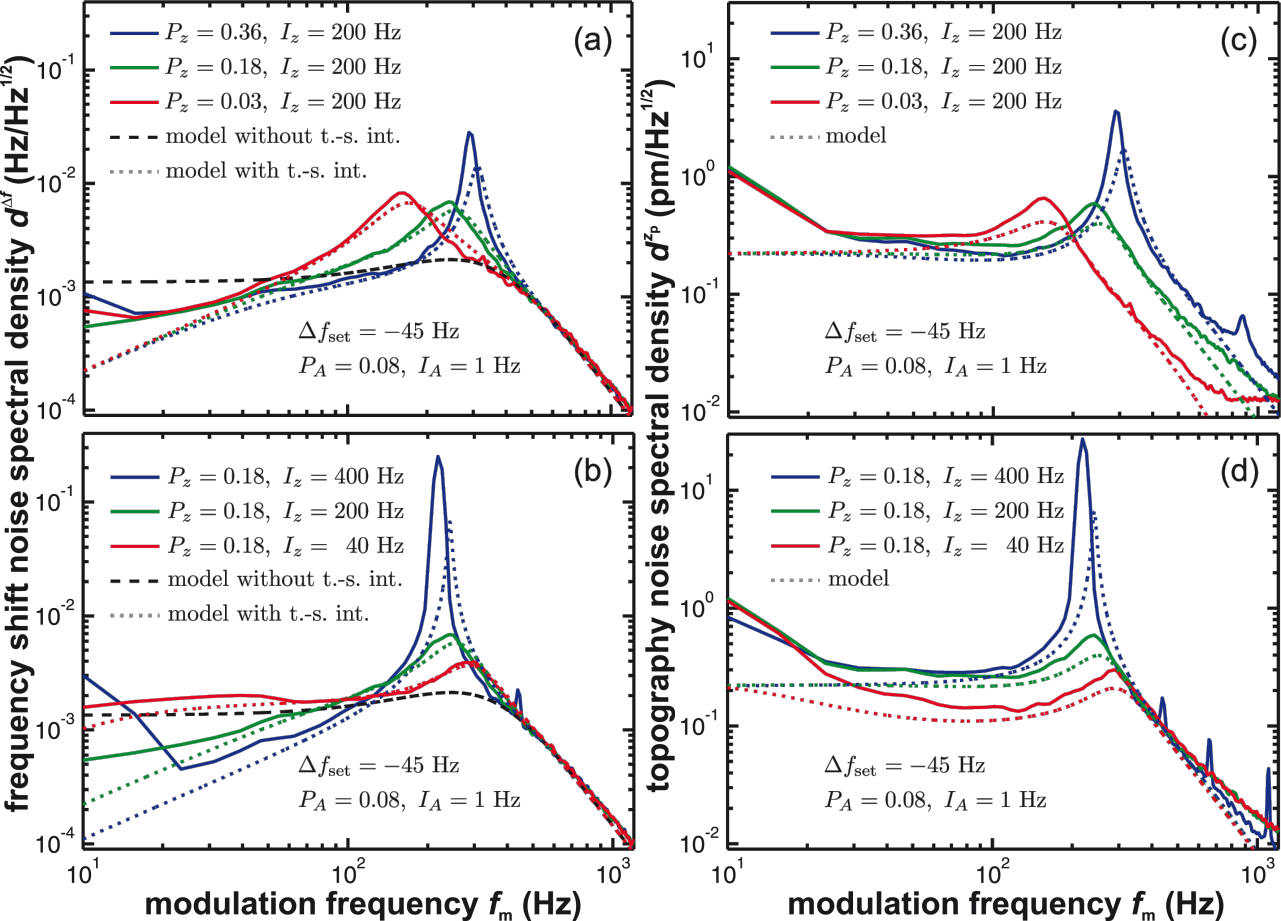
(a, b) Frequency shift noise spectral density *d*^Δ^*^f^* and (c, d) topography noise spectral density 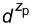 with tip–sample interaction in the constant frequency-shift mode in dependence on the distance control loop settings. Measured curves (solid lines) are compared to model predictions including tip–sample interaction (dotted lines, Equation 8 and Equation 9) and without tip–sample interaction (dashed lines, Equation 5). The loop filter *H*_lp_ has a 3rd-order Butterworth characteristics with a cutoff frequency of *f*_c_ = 500 Hz.

Finally, we investigate in Figure 8 the influence of the distance control loop parameters *P_z_* and *I_z_* on the noise characteristics. Frequency shift noise *d*^Δ^*^f^* (panels a and c) and topography noise 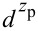 (panels c and d) experimental data (solid curves) are compared to simulations based on Equation 8 including tip–sample interaction (dotted lines) and Equation 5 without tip–sample interaction (dashed lines). In all cases, the amplitude control loop is set using optimum parameters. Data in Figure 8 are presented for different *P_z_* (*I_z_*) while keeping *I_z_* (*P_z_*) constant, respectively. Choosing the gain factors too large results in gain peaking in the noise spectral density of the frequency shift signal as well as the topography signal. Different *P_z_* (Figure 8a and Figure 8c) shift the gain peak along the frequency axis and we find in this example a minimum of the peak amplitude for *P_z_* = 0.18. This behaviour is further illustrated by discussing the frequency and step response of the distance control loop in appendix C. Decreasing *I_z_* (Figure 8b and Figure 8d) reduces the gain peaking but elevates the noise level in the low-frequency range of the frequency shift noise. In the topography noise, a decrease of *I_z_* significantly reduces the total noise. This effect is not surprising as it coincides with a reduction of the gain in the frequency range around 100 Hz and a significant slow-down of the step-response as shown in Figure 12 of appendix C. A small response time of the topography feedback loop causes a reduced noise in 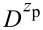.

## Conclusions and System Optimisation

We realise that the control and data acquisition system of a NC-AFM is a complex network of sensing, amplification and processing stages as well as several control loops interacting with each other. Our network analysis demonstrates the quantitative description of all frequency response functions of the NC-AFM system, including the prediction of noise confirmed by an excellent agreement between measurement and network modelling. This analysis especially provides experimental evidence for strong noise amplification by coupling of control loops due to the tip–sample interaction.

In regular NC-AFM operation with state-of-the-art hardware, signal generation and noise amplification is governed by the tip–sample interaction, which introduces the most non-linear transfer function into the system. Therefore, the optimisation of NC-AFM measurements by proper settings for system parameters is not straightforward and has to be carefully adapted to the specific measurement task. Often, corrections are necessary during measurements upon a change in tip–sample interaction, for instance due to a change in tip–sample distance or a tip change. In such situations, best results are commonly obtained by following the instinct of the experienced experimentalist.

However, the basic adjustment of the system to yield the optimum in stability, accuracy and signal-to-noise ratio can be done by a rational, systematic approach following the findings described in this paper, provided the measurement system is well characterised and offers sufficient choice and flexibility in system parameter settings.

The starting point is always the experimental task defining the desired spatial resolution λ that is, for instance, a fraction of the atomic periodicity in atomic resolution imaging, and the available time for the measurement expressed by the scan speed *v*_scan_. Assuming perfectly band-limited output signals, the sampling theorem requires the product of scan speed and inverse spatial resolution to be smaller than half of the detection bandwidth Δ*f*_BW_, or

[10]
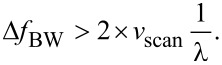


This often requires a compromise as using the optimum bandwidth defined by operation at the thermal noise limit [11] may impose a scan speed that is not practical, specifically if thermal drift is not compensated [24]. Considering the interdependence of the control loops and the tip–sample interaction, we suggest four optimisation steps to be performed in following order: (1) the PLL demodulator *H*_filter_, (2) the frequency control loop *H*_f_, (3) the amplitude control loop *H*_A_ and (4) the distance control loop *H*_z_.

In step (1), the PLL demodulator *H*_filter_ is optimised purely from simulating the frequency response to have a certain bandwidth Δ*f*_BW_. For an integral cutoff *I*_PLL_ of the PLL loop given from the ratio *f*_0_/*Q*_0_ [25] and for a low-pass filter *H*_lp_ selected according to the requested bandwidth Δ*f*_BW_, the feedback gain parameter *P*_PLL_ is increased until the peak threshold of 0.1 dB is reached (see appendix C.2 for an example and further details). As it is most desirable to work with high-*Q* cantilevers [11], the frequency control loop *H*_f_ is in step (2) inherently optimised from step (1) (see appendix C.2 for details). In case of small *Q* values (i.e., in liquid environment [16]), the optimisation in step (2) can be performed from simulating the frequency response with the knowledge of the system parameters *f*_0_ and *Q*. Optimising the amplitude control loop response *H*_A_ in step (3) requires the cantilever parameters *f*_0_ and *Q*_0_ that can easily be determined [14]. Here, the integral cutoff *I*_A_ of the amplitude loop is again set to *f*_0_/*Q*_0_ and the feedback gain parameter *P*_A_ is then increased until the threshold of 0.1 dB for gain peaking is reached as outlined in appendix C.1. This optimisation can also be performed purely from simulating the frequency response function. In step (4), the frequency response of the distance control loop is optimised. This requires the acquisition of a Δ*f*(*z*_p_) curve, from which the slope β_ts_ is calculated at the working point. The feedback loop gains *P_z_* and *I_z_* are optimised until an acceptable overshoot and a fast step response is achieved as outlined in appendix C.3. Due to the usually imminent risk of tip changes, it is advisable to plan with a safety buffer regarding these two parameters.

Specifically the last step is most crucial and requires utmost care, not only in experiment preparation but also during the experimental run. Following the outlined procedure will yield the best possible result. If this is not satisfactory, the reason is often that the base value of 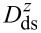 is too high or that the detection system noise contains disturbing signals, such as radio frequency interference or spurious cantilever excitation. Therefore, it is always good practice to additionally check the measurement signal with a spectrum analyser from the pre-amplifier all way down to the PLL output. The quality of measurements may dramatically be increased by removing even a minute spurious signal generated at a critical frequency to avoid its amplification by the system network. In this case, our optimisation procedure can bring the NC-AFM setup to noise-optimised performance.

## Appendix

### A Glossary

Table 1 is a glossary of all symbols used within this work to parametrise noise in an NC-AFM system.

**Table 1:** Glossary of symbols used within this work.

Function arguments	

*f* = ω/2π	frequency
*f*_m_ = ω*_m_*/2π	modulation frequency measured relative to *f*_r_
*s* = σ + *i*ω	complex frequency variable

Frequency response functions	

*H*_0_(*i*ω)	cantilever frequency response function
*H*_c_(*i*ω*_m_*)	cantilever frequency response function approximated around *f*_0_
*H*_filter_(*i*ω*_m_*)	frequency response of the PLL system
*H*_lp_(*i*ω*_m_*)	frequency response of the low-pass filter in the amplitude measurement
*H*_ac_(*i*ω*_m_*)	frequency response of the amplitude controller
*H*_zc_(*i*ω*_m_*)	frequency response of the distance controller
*H_A_*(*i*ω*_m_*)	frequency response of the amplitude control loop
*H_z_*(*i*ω*_m_*)	frequency response of the topography control loop
*H_f_*(*i*ω*_m_*)	frequency response of the frequency control loop

Cantilever and tip–sample interaction properties	

*f*_0_	modal eigenfrequency of the cantilever (fundamental mode)
*f*_r_	resonance frequency of the cantilever
*k*_0_	modal stiffness of the cantilever (fundamental mode)
*Q*_0_	modal quality factor of the cantilever (fundamental mode)
*A*_exc_	cantilever drive signal amplitude
*f*_exc_	cantilever drive signal frequency
Δ*f*_set_	frequency shift set-point
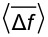	measured average frequency shift
*A*	cantilever oscillation amplitude
*A_z_*	cantilever oscillation amplitude perpendicular to the sample surface
*A*_set_	cantilever oscillation amplitude set-point
α_ts_	parameter describing the coupling between the amplitude control loop and the frequency control loop
β_ts_	parameter describing the coupling between the distance control loop and the frequency control loop

System setup parameters	

*z_p_*	scanner piezo position (topography signal)
*z*_ts_	lower turning point of the cantilever oscillation relative to the sample surface
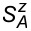	sensitivity of the cantilever deflection and the detection system
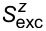	sensitivity of the cantilever excitation piezo
*S*^Δ^*^f^*	Δ*f* output signal voltage encoding of the PLL system
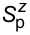	sensitivity of the *z* piezo
*f*_c_	cutoff frequency of the loop filter in the amplitude and in the frequency control loop
*P*_PLL_	proportional loop gain of the PLL
*I*_PLL_	integral cutoff of the PLL
*P_A_*	proportional loop gain of the amplitude control loop
*I_A_*	integral cutoff of the amplitude control loop
*P_z_*	proportional loop gain of the distance control loop
*I_z_*	integral loop gain of the distance control loop

Spectral densities	

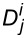	power spectral density of noise type *i* due to noise source *j*
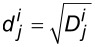	amplitude spectral density of noise type *i* due to noise source *j*
*D^z^*	total displacement noise power spectral density
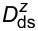	displacement noise power spectral density generated by the detection system
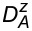	noise power spectral density describing amplitude fluctuations in the cantilever displacement signal
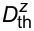	displacement noise power spectral density due to the cantilever thermal excitation
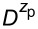	topography noise power spectral density
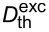	excitation noise power spectral density, describing the thermal excitation of the cantilever
*D^A^*	amplitude noise power spectral density
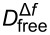	frequency shift noise power spectral density at the PLL output for the case of negligible tip–sample interaction
*D*^Δ^*^f^*	frequency shift noise power spectral density at the PLL output

### B Frequency response of control loops

We briefly outline how we calculate a closed loop response *H*_xy_ for a loop containing frequency response functions *H*_i_ between the input signal *X* and the output signal *Y*. For a more detailed discussion we refer to [26]. All frequency response functions are treated as a function of the complex frequency *i*ω. In the main text, we mostly evaluate the real component with respect to *f* = ω/(2π) (or *f*_m_) as this “amplitude response” or “gain” can directly be compared to experimental data. Furthermore, we usually do not consider the signal phase in this work, as we are interested in noise that is a result of stochastic processes. However, the frequency response functions *H*_i_(*i*ω) are treated as transfer functions *H*_i_(*s*) using the complex frequency variable *s* = σ + *i*ω to calculate step responses from an inverse Laplace transformation.

**Figure 9:**
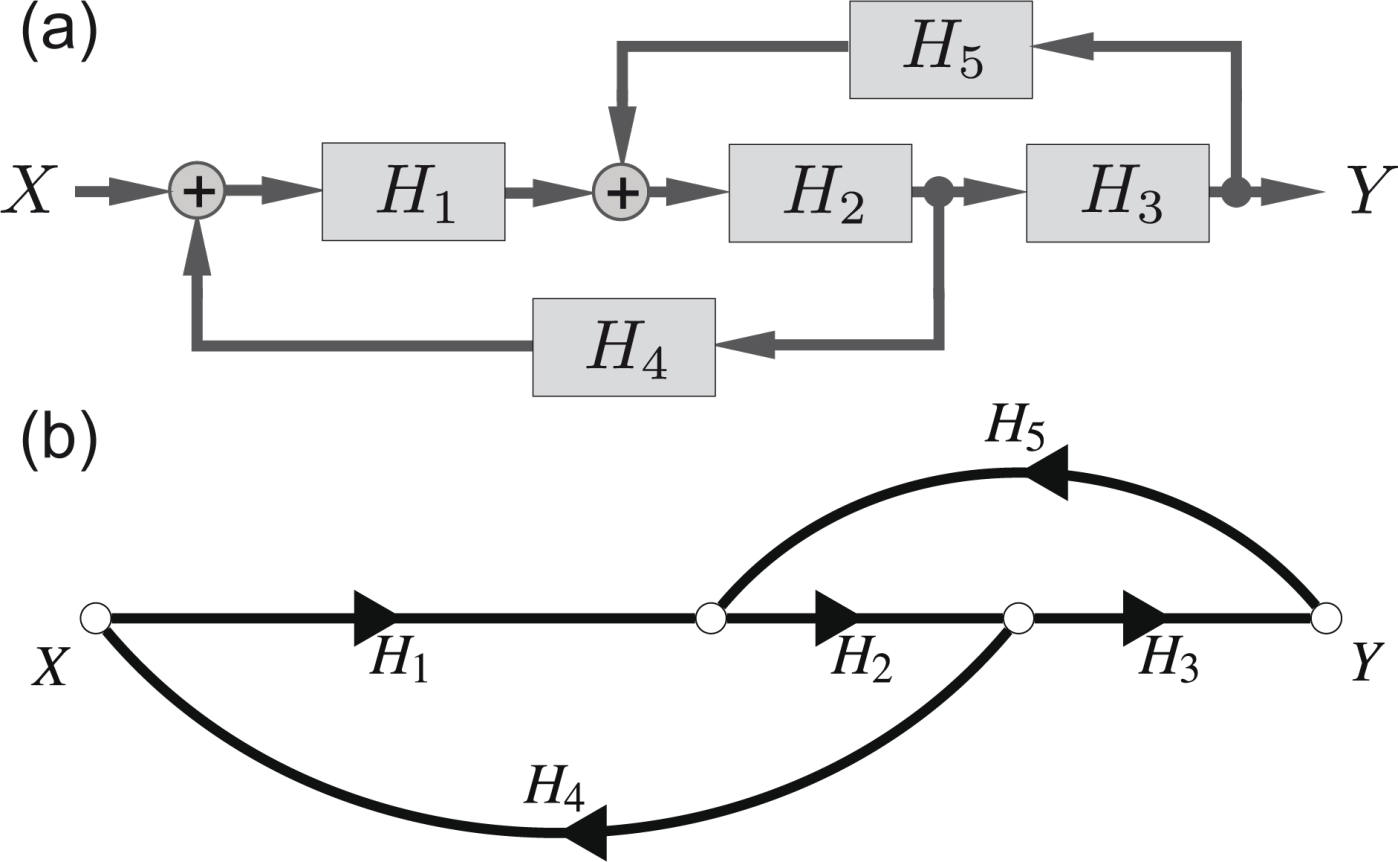
(a) Block diagram of interlaced control loops as introduced in Figure 2 and (b) signal-flow graph to demonstrate the derivation of the frequency response of coupled closed loops.

Figure 9a is a block diagram of the frequency and distance control loop of Figure 2. The model contains two closed loops that are interlaced. Using the corresponding signal-flow graph in Figure 9b and Mason’s theorem [26], we are able to describe the interlaced feedback loops by one transfer function. While the block diagram in Figure 9a focuses on the involved transfer functions, the signal-flow graph in Figure 9b represents the topological structure of the system. After using basic signal-flow graph algebra [26] and following the analysis introduced by Shinners [26], this signal-flow graph directly permits to derive a solution for the transfer function.

According to Mason’s theorem, the general expression for the signal-flow graph frequency response *H*_xy_ is

[11]
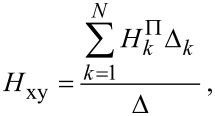


where Δ is the determinant of the graph defined as

[12]
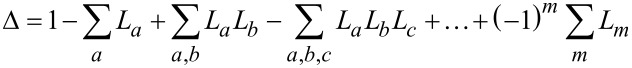


with


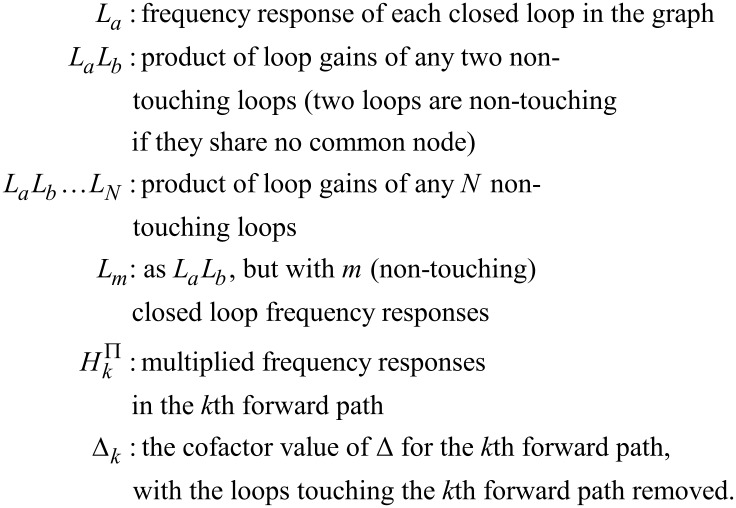


We exemplarily calculate the full frequency response for the system sketched in Figure 9 where only one forward path (*H*_1_*H*_2_*H*_3_) is in the corresponding signal-flow representation. Therefore, the calculation reduces to determine 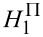 and Δ_1_. Furthermore, two non-touching closed loops, namely *H*_1_*H*_2_*H*_4_ and *H*_2_*H*_3_*H*_5_, are present. Consequently, Δ is reduced to 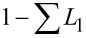 and reads

[13]
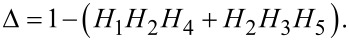


Evaluating

[14]
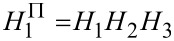


and

[15]
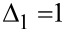


allows us to determine the full frequency response from *X* to *Y* from Figure 9 as

[16]
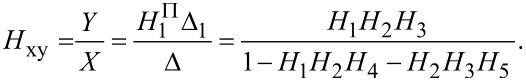


If a noise power spectral density *D_x_* is used as the input signal *X* and treated by the system response *H*_xy_ we find

[17]
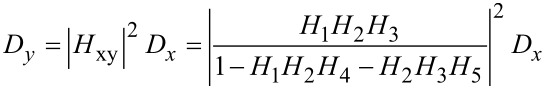


for the output noise power spectral density *D_y_* [27].

### C Frequency response functions

In this section, we present the explicit form of the frequency response functions and the specific frequency response functions valid for the experimental setup used for this work. The derivation follows [11] and [12].

#### C.1 Amplitude control loop

The frequency response *H*_ac_ of the amplitude controller is [25]

[18]
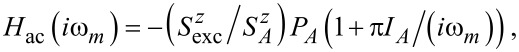


using the amplitude and excitation calibration factors 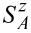 and 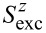, respectively, where 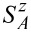 is determined by an amplitude calibration as described in [19] while 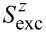 is determined from measuring the oscillation amplitude in resonance for a given excitation voltage *V*_exc_. Assuming *Q*_0_ = *A*/*A*_exc_ = 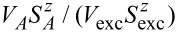 allows a straightforward calculation of 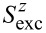 from known parameters of a well-characterised system. Note that by rewriting this formula to 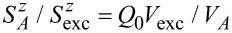, we can fully describe *H*_ac_ without performing an amplitude calibration measurement. The characteristics of the loop are defined by two parameters, the gain *P_A_* and the integral cutoff *I_A_*. Assuming that the cantilever is a system of first order, the integral cutoff *I_A_* can be chosen to *f*_0_/*Q*_0_ to avoid loop instabilities [25]. Therefore, the formula is written directly in terms of the integral cutoff and not using the integral loop gain *I* = *I_A_P_A_*π.

The frequency response of the cantilever follows from the response of a damped harmonic oscillator

[19]
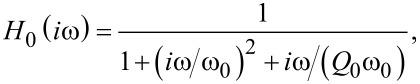


with the quality factor *Q*_0_ and the eigenfrequency ω_0_ = 2π*f*_0_. This function can be re-written with the modulation frequency ω*_m_* as the argument by substituting ω = ω_0_ + ω*_m_* and can for 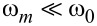 be approximated [9] to

[20]
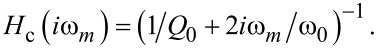


Note that Equation 20 is phase shifted by π/2 relative to Equation 19 [12]. Following procedures outlined in appendix B and Figure 2, the frequency response of the closed amplitude control loop is given as

[21]
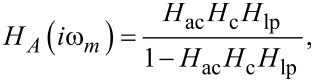


with the frequency response of the amplitude controller *H*_ac_ (see Equation 18), the frequency response of the cantilever *H*_c_ (see Equation 20) and the frequency response of the loop filter *H*_lp_, which is in our case a Butterworth filter of 3rd order [28] with cutoff frequency *f_c_*

[22]
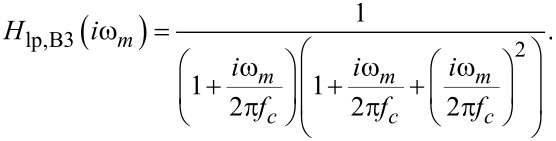


**Figure 10:**
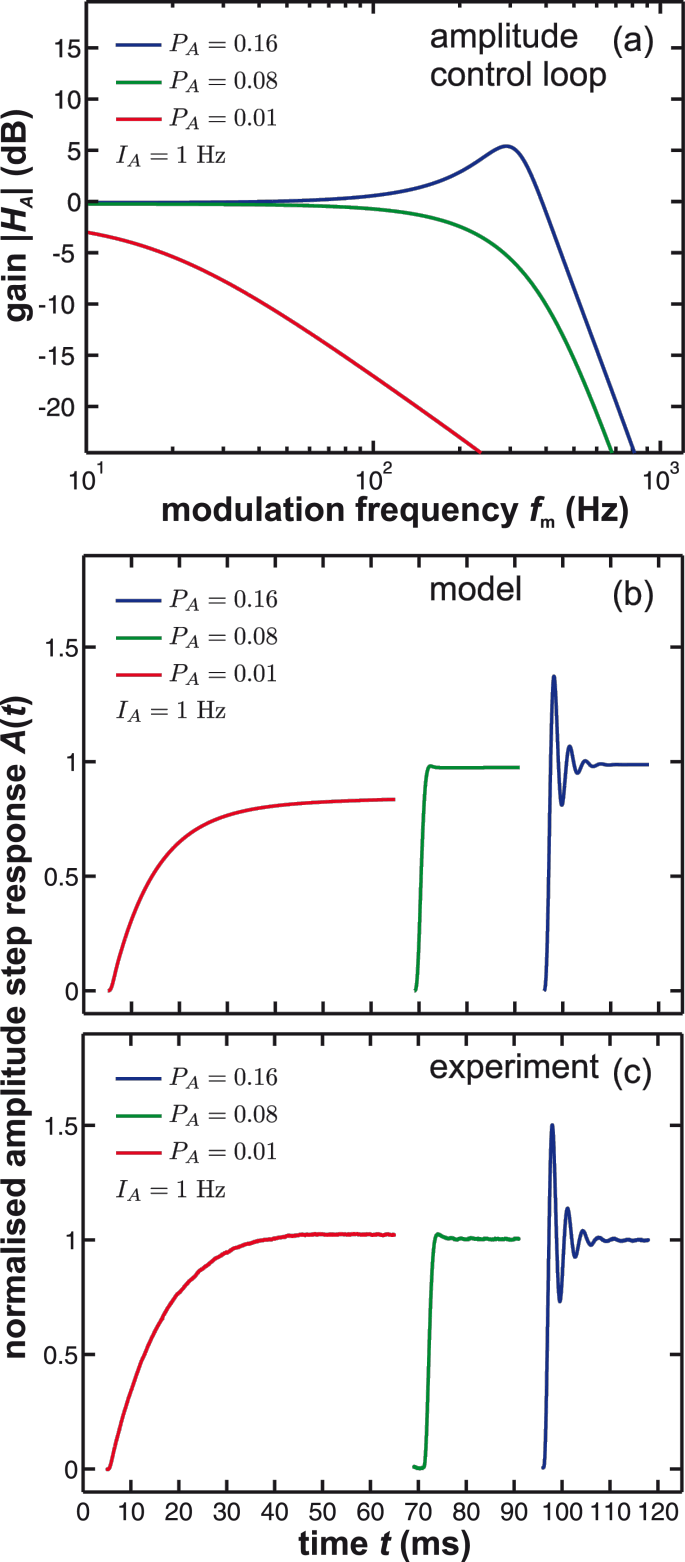
(a) Calculated gain and (b) calculated step response of the amplitude control loop compared to (c) measured step response for different loop gains *P_A_*. *I_A_* is kept fixed at 1 Hz. The loop filter *H*_lp_ has a 3rd-order Butterworth characteristics with a cutoff frequency of *f*_c_ = 500 Hz.

To quantitatively evaluate *H_A_*, we first note that *H*_c_ is fully defined by the two cantilever parameters *f*_0_ and *Q*_0_. These can easily be determined in absence of tip–sample interaction [14]. By adding the response functions of the loop filter and amplitude controller according to Equation 21, we calculate the frequency response of the amplitude control loop illustrated in Figure 10a for different proportional gain values *P_A_*. Figure 10b shows the corresponding step response in time space, which is calculated by applying the inverse Laplace transform [29] to the product of the transfer function *H_A_*(*s*) with *s* = σ + *i*ω and the Laplace transform of the unit step function, 1/*s*. The result 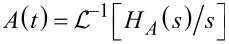 is numerically evaluated [30]. The PLLpro2 system provides a feedback test by periodically changing one parameter, here the amplitude set-point, by a given magnitude, while recording the respective response with time. The measurements are normalised to a step height of one to be comparable to the calculated step responses. As shown in panel (c), the calculations are in excellent agreement with the measured step response. The response functions follow the expected behaviour: For small *P_A_*, the frequency response is a decreasing function of frequency and the step response a slowly rising function in time (red curves). For large *P_A_*, gain peaking appears in the frequency response and the step response exhibits ringing (blue curves). The optimum setting is represented by the frequency response being flat over the low pass filter bandwidth followed by a steep decrease. This corresponds to a nearly rectangular step response with a certain rise time and a small overshoot (green curves).

To optimise the amplitude control loop parameters, we first set the integral cutoff *I_A_* of the amplitude controller to *f*_0_/*Q*_0_ [25]. To analyse the frequency response, we then start with a small *P_A_* and increase this value stepwise until a certain threshold for the gain peaking is reached, e.g., 0.1 dB. For the given set of parameters, this response reflects the optimum settings.

#### C.2 Frequency control loop

The frequency response of the PLL is given by (see Supplemental Information of [11])

**Figure 11:**
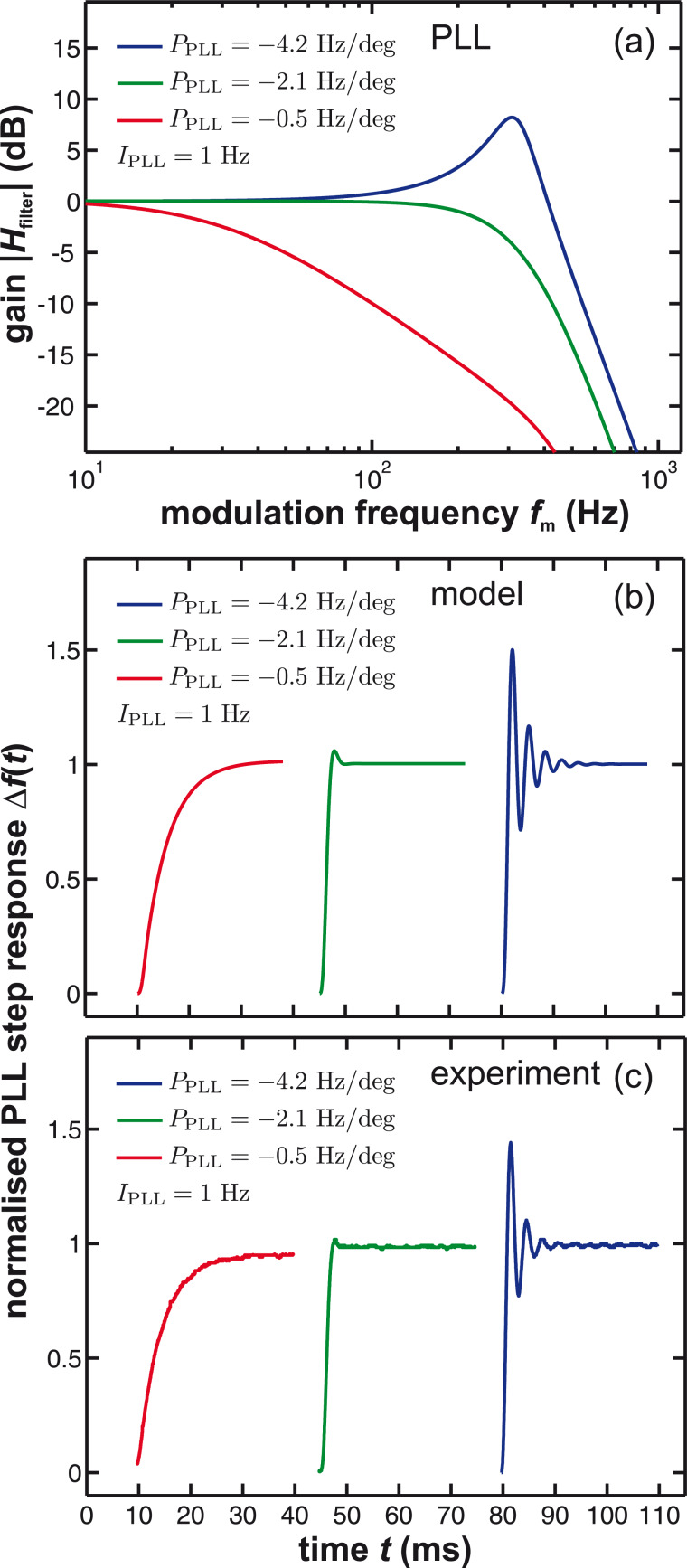
(a) Calculated gain and (b) calculated step response of the PLL compared to (c) the measured step response for different loop gains *P*_PLL_. *I*_PLL_ is kept fixed at 1 Hz. The loop filter *H*_lp_ has a 3rd-order Butterworth characteristics with a cutoff frequency of *f*_c_ = 500 Hz.

[23]
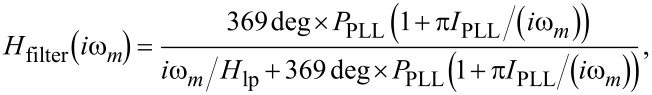


with *H*_lp_(*i*ω*_m_*) being the frequency response of the low-pass loop filter. Figure 11a illustrates the calculated gain of the PLL using Equation 23 for different loop gain settings *P*_PLL_ and a 3rd-order Butterworth filter (see Equation 22) with a cutoff frequency of 500 Hz. We experimentally observe that the integral cutoff *I*_PLL_ has a minor influence on the frequency response besides the presence of gain peaking for very small values. In Figure 11b, the step response of *H*_filter_ is calculated and compared to the measured step response of the PLLpro2 system shown in Figure 11c. Here, the inverse Laplace transform is used to calculate the frequency shift 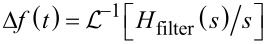 and the PLLpro2 feedback test is experimentally performed by periodically changing the phase setpoint within the PLLpro2 frequency control loop while logging the frequency shift signal.

For the closed frequency control loop, we find from Figure 2 and using procedures outlined in appendix B

[24]
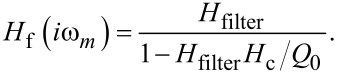


By using Equation 20, we find *H*_c_/*Q*_0_→0 for large *Q*_0_. As high *Q*_0_ values are always desirable in experiments performed under UHV conditions, this analysis suggests an optimisation procedure for the frequency control loop solely based on the frequency response *H*_filter_ of the PLL. This optimisation is possible from calculating the gain before the cantilever is inserted into the system if the system parameters *f*_0_ and *Q*_0_ are known. The procedure can be performed similar to the optimisation of the amplitude loop, by first setting the integral cutoff *I*_PLL_ to *f*_0_/*Q*_0_ and then increasing *P*_PLL_ until the threshold of 0.1 dB for gain peaking is reached. Calculated and experimentally measured frequency and step response functions are acquired as before in case of the amplitude control loop and are presented in Figure 11.

#### C.3 Distance control loop

The the *z*-distance controller is a general proportional–integral regulator with frequency response

[25]
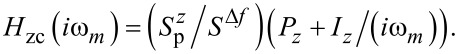


The voltage output of the PLL and the signal input of the piezos are both scaled in units of volts. To account for the correct unit of the frequency response function, we include a calibration factor *S*^Δ^*^f^* (in units of Hz/V) for the PLL output and a calibration factor 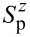 (in units of nm/V) as *z*-piezo sensitivity.

The frequency response of the closed distance control loop is determined from Figure 2 and using procedures outlined in appendix B

**Figure 12:**
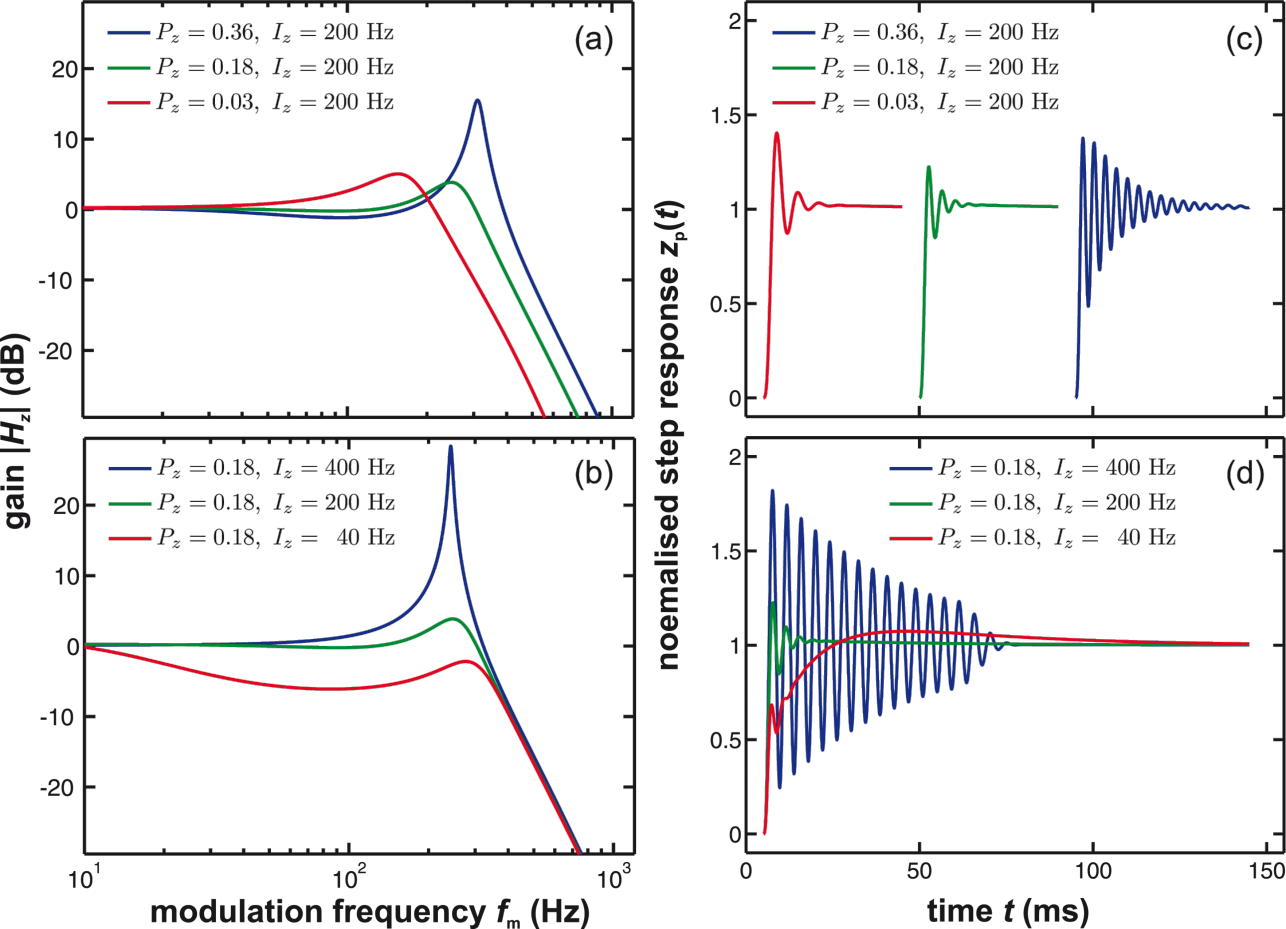
(a, b) Frequency response and (c, d) step response of the distance control loop for a given tip–sample interaction β_ts_ = 12.3 Hz/nm and different settings of *P_z_* and *I_z_*. The calculations are performed using the ratio 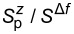 = −0.312 nm/Hz and for PLL settings *P*_PLL_ = −2.1 Hz/deg, *I*_PLL_ = 1 Hz with the loop filter *H*_lp_ having a 3rd-order Butterworth characteristics with a cutoff frequency of *f*_c_ = 500 Hz.

[26]
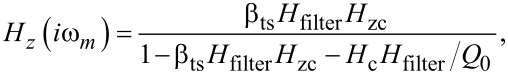


with *H*_zc_ being the frequency response of the distance controller (see Equation 25) and *H*_filter_ being the frequency response of the PLL (see Equation 23). Figure 12a,b illustrate the calculated response of the distance control loop using Equation 26 for different settings of the proportional gain *P_z_* and the integral gain *I_z_*. The corresponding calculated step response of the distance control loop is shown in Figure 12c,d.

Figure 12 illustrates that a proper adjustment of the distance controller parameters *P_z_* and *I_z_* is mandatory for stable and fast operation. Compared to the previous loop discussions, a significant complication added is the parameter β_ts_, which strongly depends on *z*_p_. Therefore, a configuration identified as the optimum for a certain tip–sample distance is most likely obsolete for stronger or weaker tip–sample interaction and would yield creep or overshoot in the step response.

For the optimisation of the distance control loop, a Δ*f*(*z*_p_) curve should be obtained first and the slope of the Δ*f*(*z*_p_) curve at the desired working point (β_ts_ = 12.3 Hz/nm, see Figure 4) should be used to simulate the frequency response of the distance control loop. As shown in Figure 12a and Figure 12c, an optimum for the lowest gain peaking could be found for *P_z_* = 0.18 (green curve). When changing the integral gain *I_z_* (see Figure 12b and Figure 12d), we start in this case from a strongly damped response (red curve), pass the optimum (*I_z_* = 200 Hz, green curve) and arrive at a ringing behaviour (blue curve). The optimum is characterised by acceptable overshoot. A fast step response is obtained by reducing gain peaking while maintaining a flat response at low frequencies. However, operating on a slightly different position on the Δ*f*(*z*) curve may strongly change the frequency response of the distance control loop. Therefore, it is advisable to plan with a safety buffer regarding the choice of *P_z_* and *I_z_* values to be prepared for unexpected changes of the tip–sample interaction.

### D Relation between α_ts_ and β_ts_

The theory derived by Polesel-Maris et al. [12] describes the impact of the tip–sample interaction on the measurement signal noise by the two parameters α_ts_ and β_ts_ defined as

[27]
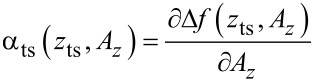


and

[28]
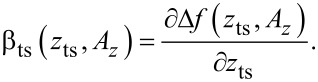


Here, *z*_ts_ denotes the *z*-position of the lower turning point of the cantilever oscillation (see Figure 3). This position is related to the piezo position *z*_p_ and the oscillation amplitude *A_z_* by

[29]
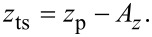


Following an amplitude change of δ*A*, the lower turning point shifts to *z*_p_ − δ*A* while the centre of the oscillation *z*_p_ remains fixed. As a consequence of Equation 29, we find an identity for the derivations with respect to *z*_ts_ and *z*_p_:

[30]
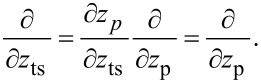


Using this identity and Equation 29, we rewrite Equation 27 and Equation 28:

[31]
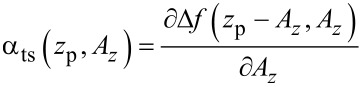


[32]
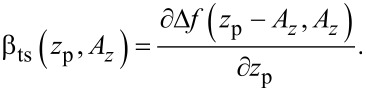


Thus, the parameter β_ts_ can be determined directly from the slope of a known Δ*f*(*z*_p_) curve as shown in Figure 4. Furthermore, Equation 31 can be rewritten into two terms

[33]
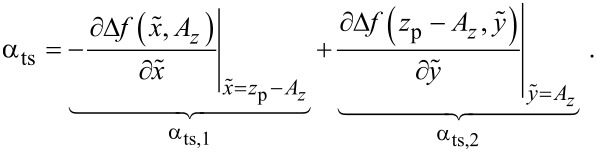


This representation explicitly presents the two effects of an amplitude change on the frequency shift: First, the frequency shift changes due to a different lower turning point (α_ts,1_) and, second, the change in Δ*f* due to a change of the weighting function [21] in the Δ*f* calculation (α_ts,2_).

The first term is a measure of the slope of the Δ*f*(*z*_p_) curve with respect to the piezo position *z*_p_. It is identical to −β_ts_. The second term is a result from the convolution of the tip–sample force interaction with a weighting function [21]. For large oscillation amplitudes, a dependence 
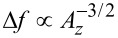
 has been found, allowing the definition of an amplitude-independent, normalised frequency shift [21]. This second term becomes negligible for large oscillation amplitudes *A_z_*.

We illustrate the latter point by using an analytic expression for a Morse interaction force

[34]
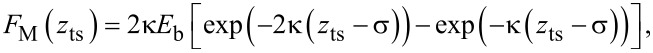


for which the resulting frequency shift Δ*f*_M_ can be calculated as [31]

[35]
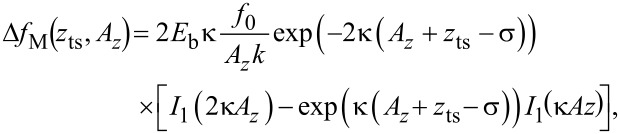


where *I_n_*(*z*) is the modified Bessel function of the first kind. Using this expression, parameters α_ts_ and β_ts_ are directly calculated as

[36]
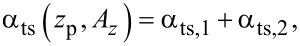


[37]
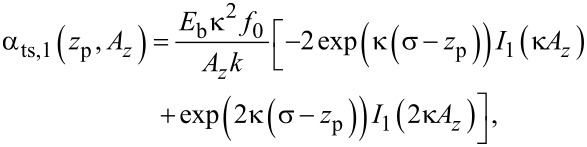


[38]
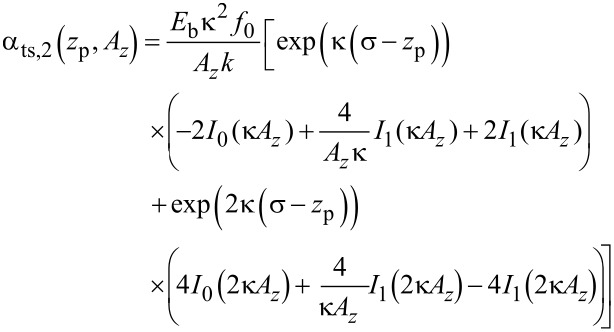


[39]
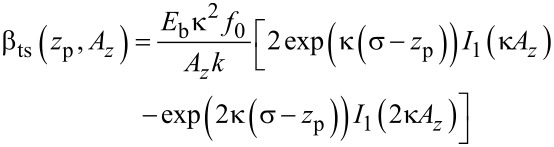


The statement α_ts,1_ = −β_ts_ is directly evident from Equation 37 and Equation 39. To quantify the relation between α_ts,1_ and α_ts,2_, we plot the ratio

**Figure 13:**
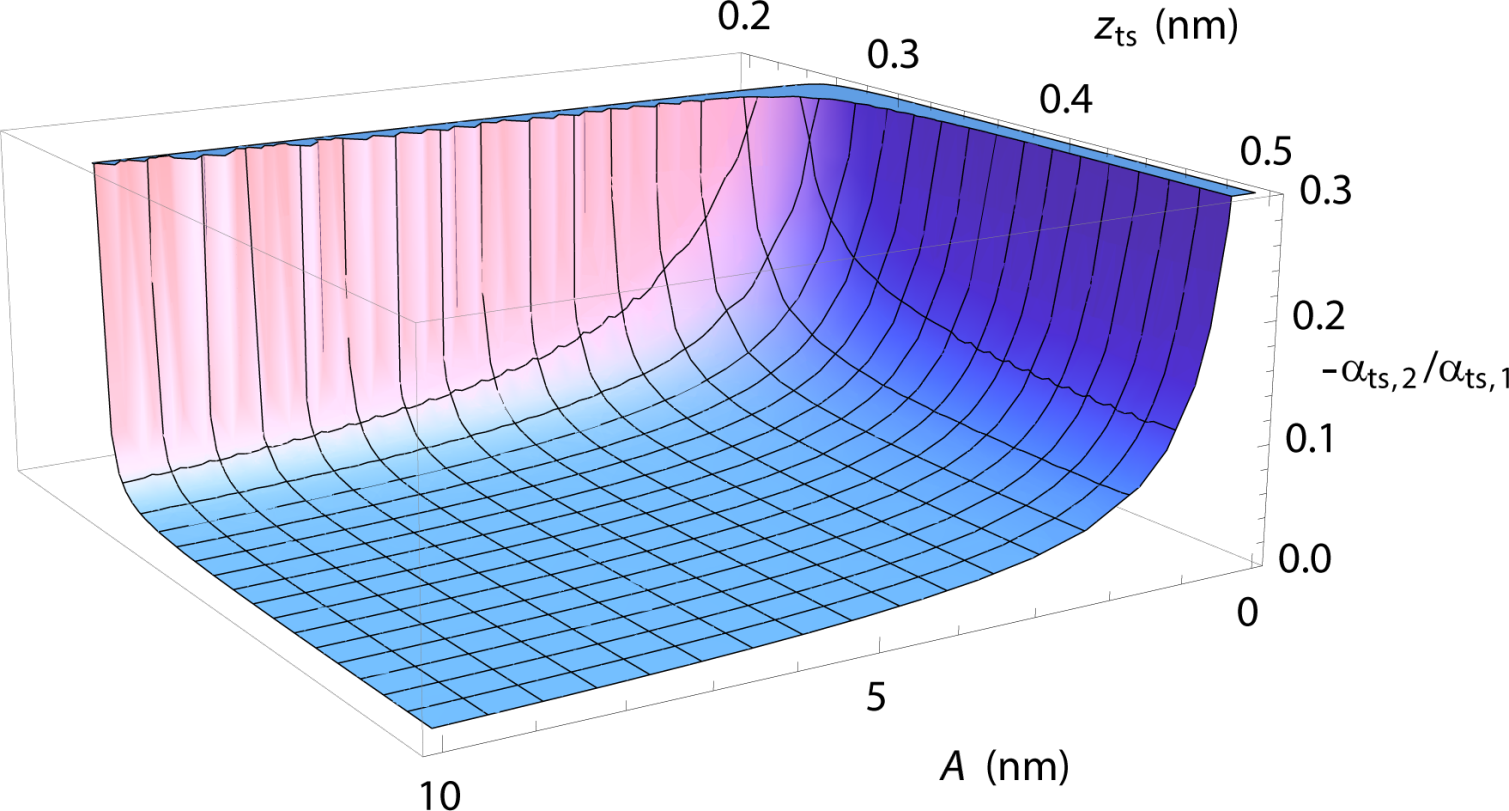
Ratio δα = −α_ts,2_/α_ts,1_ as a function of the *z*-position and the amplitude. A Morse interaction using parameters from [32] and *f*_0_ = 300 kHz, *k* = 35 N/m are used to model the tip–sample interaction.

[40]
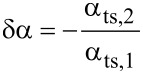


as a function of the amplitude *A* and the lower turning point position *z*_ts_ in Figure 13 and using parameters for a Si–Si interaction derived from theory [32], namely *E*_b_ = 2.273 eV, κ = 12.76 nm^−1^ and σ = 0.2357 nm. Even at *z*-positions close to the force minimum (*z*_min_ ≈ 0.3 nm) and for amplitudes *A* larger than 5 nm, the parameter α_ts,2_ is less than 5% of α_ts,1_. Thus, under these conditions, the approximation

[41]
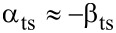


is fully justified.

## Supporting Information

File 1 Scripts implementing all transfer functions and noise signals defined within this article.
